# Massively Parallel CRISPR‐Based Genetic Perturbation Screening at Single‐Cell Resolution

**DOI:** 10.1002/advs.202204484

**Published:** 2022-12-11

**Authors:** Junyun Cheng, Gaole Lin, Tianhao Wang, Yunzhu Wang, Wenbo Guo, Jie Liao, Penghui Yang, Jie Chen, Xin Shao, Xiaoyan Lu, Ling Zhu, Yi Wang, Xiaohui Fan

**Affiliations:** ^1^ Pharmaceutical Informatics Institute College of Pharmaceutical Sciences Zhejiang University Hangzhou Zhejiang 310058 China; ^2^ State Key Laboratory of Component‐Based Chinese Medicine Innovation Center in Zhejiang University Hangzhou 310058 China; ^3^ Jinhua Institute of Zhejiang University Jinhua 321016 China; ^4^ The Save Sight Institute Faculty of Medicine and Health the University of Sydney Sydney NSW 2000 Australia; ^5^ Future Health Laboratory Innovation Center of Yangtze River Delta Zhejiang University Jiaxing 314100 China; ^6^ Westlake Laboratory of Life Sciences and Biomedicine Hangzhou 310024 China

**Keywords:** CRISPR/Cas, genetic screening, imaging‐based phenotyping, multiplexed perturbations, single‐cell sequencing

## Abstract

The clustered regularly interspaced short palindromic repeats (CRISPR)‐based genetic screening has been demonstrated as a powerful approach for unbiased functional genomics research. Single‐cell CRISPR screening (scCRISPR) techniques, which result from the combination of single‐cell toolkits and CRISPR screening, allow dissecting regulatory networks in complex biological systems at unprecedented resolution. These methods allow cells to be perturbed en masse using a pooled CRISPR library, followed by high‐content phenotyping. This is technically accomplished by annotating cells with sgRNA‐specific barcodes or directly detectable sgRNAs. According to the integration of distinct single‐cell technologies, these methods principally fall into four categories: scCRISPR with RNA‐seq, scCRISPR with ATAC‐seq, scCRISPR with proteome probing, and imaging‐based scCRISPR. scCRISPR has deciphered genotype–phenotype relationships, genetic regulations, tumor biological issues, and neuropathological mechanisms. This review provides insight into the technical breakthrough of scCRISPR by systematically summarizing the advancements of various scCRISPR methodologies and analyzing their merits and limitations. In addition, an application‐oriented approach guide is offered to meet researchers’ individualized demands.

## Graphical abstract

Single‐cell clustered regularly interspaced short palindromic repeats (CRISPR)‐based genetic perturbations screening (scCRISPR) enables investigation of the complex genetic regulatory systems and provides insights into the cell heterogeneity in genetic perturbations. Based on the diversity of scCRISPR readout, scCRISPR methods can be categorized into four types: scCRISPR with RNA‐seq, scCRISPR with ATAC‐seq, scCRISPR with proteome probing, and imaging‐based scCRISPR.

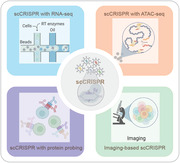

## 1 Introduction

CRISPR (clustered regularly interspaced short palindromic repeats) technology is an indispensable tool for functional genomics research.^[^ ^1^ ^]^ CRISPR, which consists of the nuclease (Cas), the crRNA (CRISPR RNA), and the tracrRNA (transactivating crRNA; crRNA and tracrRNA are typically synthesized as a single guide RNA; sgRNA), enables more efficient and precise gene manipulation, including ablation, activation, substitution, base transversion, and even epigenomic modification, than traditional gene‐editing methods such as transcription activator‐like effector nuclease.^[^ ^2^ ^]^

**Figure 1:**
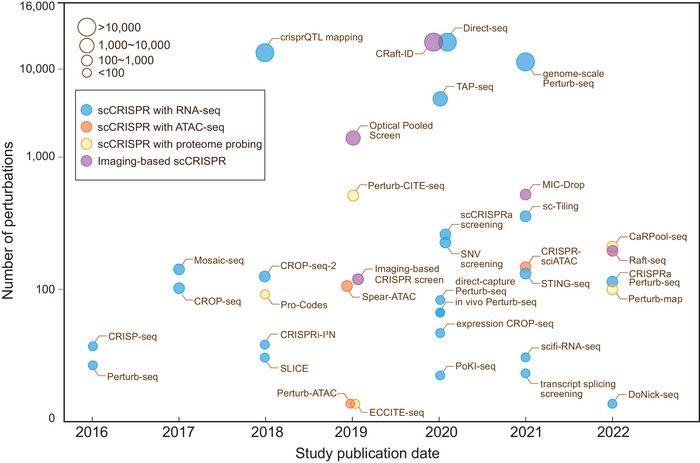
Advancement of scCRISPR approaches. Over the past 6 years, scCRISPR has continued to evolve and can be divided into four main categories based on the single‐cell technologies that are combined (as indicated in different colors). These scCRISPR methods play an irreplaceable role in large‐scale genetic perturbation screening to investigate functional genomics and have the potential to yield significant biological discoveries (the number of perturbations shown in the figure is the number of each CRISPR technology in its original publication and does not completely represent the screening capacity of each technology).

CRISPR‐based genetic screens, which examine the cellular changes following an intended genetic perturbation, have become a widely‐utilized method for investigating gene functions and molecular mechanisms.^[^ ^3^ ^]^ Cells are routinely delivered a library of hundreds of sgRNAs at a low viral concentration to ensure that each cell receives a single sgRNA. The gene's contribution to overcoming adversity can be evaluated by sequencing sgRNAs frequency in viable cells after the cell mosaic has been cultured with selective challenges. However, this assay just yields a singular readout and cannot meet the needs of complex mechanical research. In contrast, arrayed screens where physically separated cells are individually perturbed with distinct virus packages allow obtaining high‐content phenotypes and accurately validating gene functions but are laborious and non‐scalable. In the past 6 years, single‐cell CRISPR screening (scCRISPR) arose to be a hybrid technique with the potential to overcome the limitations of pooled and arrayed screens. These techniques combine the efficiency and flexibility of CRISPR tools with single‐cell platforms, such as scRNA‐seq, single‐cell assays for transposase‐accessible chromatin with sequencing (scATAC‐seq), cellular indexing of transcriptomes and epitopes by sequencing (CITE‐seq), and single‐cell imaging, to form a grand genealogy (**Figure** **1**).^[^ ^4^ ^]^ Some techniques even allow for the simultaneous perturbation analysis of thousands of genes. scCRISPR screening currently renders both high‐content readout and high‐throughput genetic perturbation.

Technically, the critical parameter of scCRISPR is recalling sgRNA identity after characterizing single‐cell profiles^[^ ^5^ ^]^ to correctly annotate each cell with genotype information. The pioneering techniques (Perturb‐seq, CRISP‐seq, Mosaic‐seq) track sgRNA identity by inserting unique guide barcodes (GBC) into sgRNA‐encoding plasmids.^[^ ^6^ ^]^ The endogenous mRNA and GBC transcripts are ligated by the same cell barcode (CBC) during reverse transcription (RT) within the built‐in microchambers of single‐cell platforms. Through the mapping of two barcodes within single sequencing reads, transcriptomes and corresponding perturbations can be related. Beyond DNA barcoding, branching detection strategies of sgRNA have arisen during the past 6 years, diversifying the screening modes of scCRISPR including genetic knock‐in and spatially resolved readout.^[^ ^5^, ^7^ ^]^ The swift evolution in scCRISPR potentiates its applications in not only genetic screening, but also genetic profiling that substantiates our knowledge in fundamental biology, tumor behavior, and translational medicine.

To date, 36 scCRISPR approaches have been developed to cover diverse specialized capabilities and have been applied in additional 30 applications in the genetic biology frontier. However, there are still gaps and deficiencies in its technological realm. To facilitate the development of scCRISPR, this review examines how distinct single‐cell platforms harmonize with CRISPR toolkits referring to their technical details, highlighting their merits and limitations and diverse applications. We underline the perspective from which each approach inherits and innovates from its precursor and how these methods differ. Then, we discuss their prospective applications in imaging‐based genotype‐to‐phenotype mapping, genetic regulation analysis, tumor biology, and neurological issues. Given the extensive versatility of scCRISPR techniques, we propose a programmable, demand‐oriented decision tree for researchers seeking to make preliminary decisions. The analysis of the high‐content scCRISPR readout data is summarized and the challenges posed by the high‐dimensional data are also highlighted. Finally, we summarize and make prospects on some challenging problems in the field of scCRISPR screening, and try to make an extensive discussion on its possible role in addressing puzzles from biological genomics and medicine.

## 2 Advances in scCRISPR Technologies

**Figure 2:**
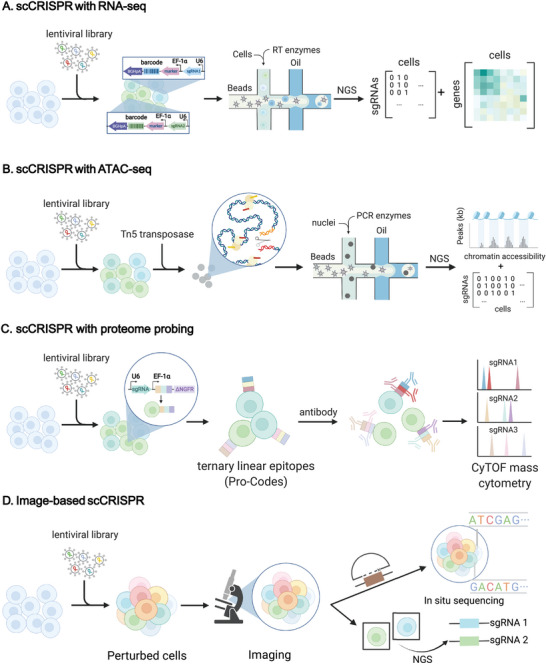
scCRISPR approaches. A) scCRISPR with RNA‐seq, exemplified by Perturb‐seq. Pooled Cas9‐expressing cells are transduced with a barcoded lentiviral library at a proper MOI. After filtration of successfully transduced cells with a biological challenge or flow cytometry, most remaining cells each received one perturbation. The non‐polyadenylated sgRNAs which are driven by the U6 promoter and transcribed by Pol III, work with the CRISPR effector to conduct gene editing. Driven by the EF‐1*α* promoter, the sgRNA‐specific barcode and reporter's co‐transcript will be polyadenylated. After cells are lysed in droplets generated in the microfluidic apparatus, the released sgRNA‐specific barcodes, and endogenous transcripts are simultaneously reverse‐transcribed and labeled with the same cell barcode. Finally, computational analysis of sequencing data correlates each cell's transcriptome with its perturbation identity. NGS, next‐generation sequencing; RT enzymes, reverse‐transcription enzymes; EF‐1*α*, elongation factor‐1*α*; U6, U6 promoter; NGS, next‐generation sequencing. B) scCRISPR with ATAC‐seq, exemplified with Spear‐ATAC, the sgRNA was pre‐integrated with Nextera adapters and a biotin‐tagged primer. The perturbed K562 cells were then subjected to nuclear isolation and transposition according to the modified ATAC‐seq protocol to obtain the ATAC fragments. The ATAC fragments, together with the cDNA of the sgRNA produced by targeted RT, undergo two rounds of PCR to obtain the library for sequencing. C) scCRISPR with proteome probing. In Pro‐Codes, linear epitope combinations are utilized as barcodes to probe the surface protein, and antibodies containing metal isotopes can be identified by mass cytometry. D) Imaging‐based scCRISPR. Imaging‐based scCRISPR investigates the association between genotype and spatial phenotype by capturing optical barcodes directly (e.g., by fluorescent in situ hybridization through padlock‐based methods) or by selecting cells with phenotypes of interest before identification of perturbations in single cells by targeted sequencing.

Since 2016, scCRISPR has emerged from the interdisciplinary areas of CRISPR screening and single‐cell technology. To cater to different research topics, CRISPR‐based loss‐of‐function and gain‐of‐function screens using CRISPR effectors including Cas9, dead Cas9 (dCas9), Cas9 nickase (Cas9n), Cas13d, and cytosine base editor (CBE) can be implemented on single‐cell devices, such as microfluidics, as well as flow and mass cytometry. For the critical role of single‐cell platforms in scCRISPR, this review categorizes scCRISPR methods into four types (detailed in **Table** **1**): scCRISPR with RNA‐seq, scCRISPR with ATAC‐seq, scCRISPR with proteome probing, and imaging‐based scCRISPR (**Figure** **2**). These different screening methodologies vary greatly in technical parameters such as the perturbation mechanism, sgRNA library size, number of perturbed cells, and the type of readout data (detailed in Table 1).

**Table 1:** Overview of the scCRISPR methods

Method	Perturbation mechanism	Medium of sgRNA identify	Library	No. of cells	Modularity	Single‐cell platform	Ref.
Perturb‐seq^a)^	CRISPRi	Barcode	9 tgRNAs (3 genes)	15 006	RNA	10× Chromium	[6a]
	CRISPRko	Barcode	43 sgRNAs (15 genes)	25 971	RNA	10× Chromium	[6b]
CRISP‐seq^a)^	CRISPRko	Barcode	57 sgRNAs (22 genes)	6144	RNA	MARS‐seq	[6c]
Mosaic‐seq^a)^	CRISPRi	Barcode	241 sgRNAs (71 enhancers)	12 444	RNA	Drop‐seq	[11b]
CROP‐seq^a)^	CRISPRko	Polyadenylated sgRNA	116 sgRNAs (32 genes)	5798	RNA	Drop‐seq	[7a]
CROP‐seq‐2^a)^	CRISPRko	Polyadenylated sgRNA	183 sgRNAs (29 genes)	12 881	RNA	10× Chromium	[20]
crisprQTL mapping^a)^	CRISPRi	Polyadenylated sgRNA	11558 sgRNAs (5779 enhancers)	207 324	RNA	10× Chromium	[11a]
SLICE^a)^	CRISPRko	Polyadenylated sgRNA	48 sgRNAs (20 genes)	≈15 000	RNA	10× Chromium	[23]
CRISPRi‐I^3^N^a)^	CRISPRi	Polyadenylated sgRNA	58 sgRNAs (27 genes)	≈40 000	RNA	10× Chromium	[26]
in vivo Perturb‐seq^a)^	CRISPRi	Barcode	80 sgRNAs (38 genes)	46 770	RNA	10× Chromium	[18]
PoKI‐seq^a)^	CRISPR knock‐in	Barcode	36 donor DNAs	≈40 000	RNA	10× Chromium	[16]
TAP‐seq^a)^	CRISPRi	Polyadenylated sgRNA	7055 sgRNAs (1778 enhancers)	231 667	RNA	10× Chromium/ Drop‐seq	[19]
SNV screening^a)^	CRISPR base editing	Polyadenylated sgRNA	420 sgRNAs (3 genes)	13 218	RNA	Drop‐seq	[21]
scCRISPRa screening^a)^	CRISPRa	Polyadenylated sgRNA	475 sgRNAs (230 genes)	203 894	RNA	10× Chromium	[54]
scifi‐RNA‐seq^a)^	CRISPRko	Polyadenylated sgRNA	48 sgRNAs (20 genes)	20 710	RNA	10× Chromium	[22b]
direct‐capture Perturb‐seq^a)^	CRISPRko, CRISPRi, CRISPRa	sgRNA	431 dgRNAs (104 genes)	211 103	RNA	10× Chromium	[5]
Direct‐seq^a)^	CRISPRko, CRISPRa	sgRNA	12472 sgRNA pairs	13 435	RNA	10× Chromium/Fluidigm C1	[7b]
sc‐Tiling^a)^	CRISPRko	sgRNA	602 sgRNAs (1 gene)	4943	RNA	10× Chromium	[27]
transcript splicing screening^a)^	CRISPRko	sgRNA	37 sgRNAs (16 genes)	3073	RNA	Oxford nanopore; 10× Chromium	[28]
STING‐seq^a)^	CRISPRi	sgRNA	210 sgRNAs (88 genes)	9343	RNA + protein	10× Chromium	[53a]
genome‐scale Perturb‐seq^a)^	CRISPRi	sgRNA	10673 dgRNA (9866 genes)	2 061 931	RNA	10× Chromium	[80]
DoNick‐seq^a)^	CRISPRko	Barcode	13 sgRNA quartets (13 genes)	21 657	RNA	10× Chromium	[13]
Expression CROP‐seq^a)^	CRISPRko	Polyadenylated sgRNA	67 sgRNAs (20 genes)	13 332	RNA	10× Chromium	[107]
CRISPRa Perturb‐seq^a)^	CRISPRa	sgRNA	150 sgRNAs (69 genes)	≈56 000	RNA	10× Chromium	[108]
Perturb‐ATAC^b)^	CRISPRi, CRISPRko	Barcode	14 tgRNAs (12 genes)	2627	DNA	Fluidigm C1	[29]
Spear‐ATAC^b)^	CRISPRi, CRISPRko	sgRNA in genome	128 sgRNAs (40 genes)	32 832	DNA	10× Chromium	[30]
CRISPR‐sciATAC^b)^	CRISPRko	Polyadenylated sgRNA	255 sgRNAs (84 genes)	16 676	DNA	Combinatorial indexing	[55]
Pro‐Codes^c)^	CRISPRko	Barcode	96 sgRNAs (54 genes)	500 000	Protein	CyTOF	[10c]
Perturb‐map^c)^	CRISPRko	Barcode	101 sgRNAs (35 genes)	8 442 439	Protein + RNA + imaging	CyTOF, MICSSS, MIBI, 10× Visium	[39]
ECCITE‐seq^c)^	CRISPRko	sgRNA	13 sgRNAs (4 genes)	4120	Protein + RNA	10× Chromium	[42]
Perturb‐CITE‐seq^c)^	CRISPRko	Polyadenylated sgRNA	744 sgRNAs (248 genes)	87 590	Protein + RNA	Drop‐seq, flow cytometer	[25]
CaRPool‐seq^c)^	CRISPR RNA knockdown	Barcode	385 tgRNA (26 genes)	31 308	Protein + RNA	10× Chromium	[17]
imaging‐based CRISPR^c)^ screen^d)^	CRISPRko	Polyadenylated sgRNA	162 sgRNAs (54 genes)	≈30 000	Imaging	MERFISH	[64]
Optical Pooled Screen^d)^	CRISPRa, CRISPRko	Polyadenylated sgRNA	3063 sgRNAs (952 genes)	3 037 909	Imaging	Microscopy	[44b]
CRaft‐ID^d)^	CRISPRko	sgRNA in genome	12478 sgRNAs (1142 genes)	119 050	Imaging	Microscopy	[48]
MIC‐Drop^d)^	CRISPRko	Imported spacer cDNA	752 sgRNAs (188 genes)	7887 embryos	Imaging	Pico‐injector, microscopy	[50]
Raft‐seq^d)^	CRISPRko	cDNA mutation	357 sgRNAs (1 gene)	1659	Imaging	CellRaft air system, microscope	[49]

^a)^ Indicates scCRISPR with RNA‐seq;

^b)^ Indicates scCRISPR with ATAC‐seq;

^c)^ Indicates scCRISPR with proteome probing;

^d)^ Indicates imaging‐based scCRISPR. The identity information of sgRNA (or knock‐in construct) is mainly carried by sgRNA‐specific barcode (in Perturb‐seq; “barcode” in table), polyadenylated sgRNA (in CROP‐seq), sgRNA with capture sequence (in Direct‐seq; “sgRNA” in table), and cDNA of sgRNA integrated into host genome (in Craft‐ID; “sgRNA in genome” in table). CyTOF, cytometry by time‐of‐flight.

### 2.1 scCRISPR with RNA‐seq

**Figure 3:**
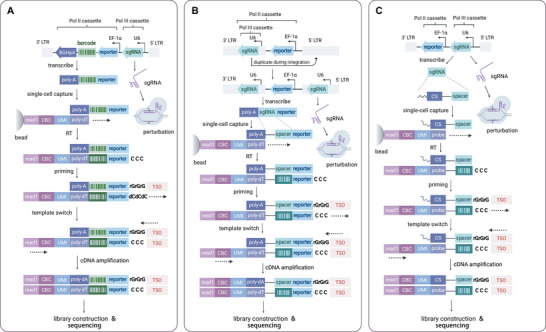
Principal strategies for detecting perturbations in scCRISPR. A) With sgRNA‐specific barcodes, exemplified by Perturb‐seq. After integration into the host genome, barcoded mRNA is transcribed from the Pol II cassette，and functional sgRNA is expressed from the Pol III cassette. In single‐cell, barcoded oligonucleotides on the gel bead can anneal to the poly(A) tail of single‐cell mRNAs. Using a 10× Chromium 3’ kit, barcodes are reverse‐transcribed into cDNAs, with three unpaired cytidines at the 5’ terminus. The TSO was added to synthesize the untruncated double‐strand DNA for further library construction and sequencing. The identities of sgRNA are deduced indirectly from reads that contain the sgRNA‐specific barcode. The quadrangles with a darker background represent the cDNA complementary to the barcode. B) With polyadenylated sgRNAs, exemplified by CROP‐seq. The sgRNA cassette (Pol III cassette) is positioned in the 3’ long terminal repeat (3’LTR), upstream of reporter genes. In this way, the sgRNA cassette is included in the reachable region of Pol II recruited by EF‐1*α*. Co‐transcription of sgRNA in the 3’LTR and reporters allows polyadenylation of sgRNA, which enables direct detection of sgRNA on poly (dT)‐supported scRNA‐seq platforms. The barcode‐like quadrangles represent the cDNA complementary to the spacer sequences. On the other hand, a copy of the sgRNA cassette will translocate into the 5’LTR during transduction, retaining the intact function of CRISPR editing. C) With sgRNAs containing the capture sequence, exemplified by 3’ direct capture Perturb‐seq. In 3’ direct capture, a specifically designed capture sequence is inserted into the loops or 3’ terminus without impairing sgRNA's function, serving as an annealing site for cDNA probes. The barcode‐like quadrangles in the figure represent the DNA complementary to spacer sequences. TSO, template switch oligo; Pol II, RNA polymerase II; Pol III, RNA polymerase III; LTR, long terminal repeat; EF‐1*α*, elongation factor‐1*α*; CS, capture sequence; CBC, cell barcode; UMI, unique molecule identifier.

In the past decade, scRNA‐seq has been the preferred method for single‐cell profiling. It offers a comprehensive understanding of gene activities and regulatory networks.^[^ ^8^ ^]^ scRNA‐seq is reliably performed on microfluidic platforms such as 10× Chromium, Drop‐seq, and Fluidigm C1, where cells are dispersed into numerous nanoliter droplets (Figure 2A). Within the single‐cell droplets, RT enzymes reversely transcribe transcripts. The complementary DNA (cDNA) is then pooled and amplified by polymerase chain reaction (PCR) in preparation for high‐throughput sequencing. As sgRNAs are generated by RNA polymerase III (Pol III) rather than RNA polymerase II (Pol II), they cannot be detected by current single‐cell devices unless they are polyadenylated. To address this problem, a Pol II‐driving cassette encoding GBC can be assembled into the plasmid vector concurrently (**Figure** **3**A). The matched barcodes can be captured immediately, enabling one‐to‐one mapping of transcriptomes to sgRNAs or sgRNA combinations.^[^ ^6^, ^9^ ^]^ However, multi‐test issues and the template‐switching effect during viral co‐packaging remain to be challenging unless the homology transfer vector or an arrayed package is utilized.^[^ ^10^ ^]^ To solve this issue, an alternate method positions the sgRNA downstream of the reporter cassettes and generates polyadenylated sgRNAs via Pol III‐driven co‐transcription of the two cassettes (Figure 3B). However, the excessive length of this nested cassette prevents multiplexed perturbation and decreases the rate of sgRNA retrieval.^[^ ^7a^ ^]^ Thus, the third strategy was designed with a capture sequence (CS) in the constant region of sgRNA (Figure 3C). In addition to the preceding strategies, targeted sequencing of cDNA of targeted sequences may also be employed on occasion (Table 1). Given that the three primary detection strategies are taken from scRNA‐seq and cover nearly all scCRISPR methods, we arranged the methods according to which sgRNA identity carrier it involves: sgRNA‐specific barcode, polyadenylated sgRNA, or sgRNA with capture sequence.

#### 2.1.1 sgRNA‐Specific Barcode

Adamson et al., who established Perturb‐seq, proposed that sgRNA identity can be retrieved by introducing and then scanning sgRNA‐specific barcodes. In a backbone plasmid, they inserted random 18‐nt barcodes between the blue fluorescent protein reporter gene and the polyadenylation signal, followed by individually cloning the sgRNA upstream (Figure 3A). Sanger sequencing was used to identify sgRNA‐barcode pairsto form an annotation reference. For multiplexed perturbation, they then designed a tandem construct of three sgRNAs. Three distinct promoters and modified constant areas were employed to minimize intramolecular recombination and ensure equal functions for each sgRNA.^[^ ^6a^ ^]^ CRISPR‐based genetic interference (CRISPRi) screens were performed on tens of genes using three‐way perturbation vectors and transcriptomes as read‐outs. In both tests, the “CBC‐GBC” segment was amplified using targeted PCR. Parallelly, Dixit et al. also developed Perturb‐seq with Cas9‐based knock‐out (CRISPRko) (Table 1). Differently, they proved that Perturb‐seq could achieve multiplexed perturbations through transducing cells at a high multiplicity of infection (MOI).^[^ ^6b^ ^]^ This strategy, common in epistasis studies or in cases when the majority of sgRNAs are non‐functional,^[^ ^11^ ^]^ is dispensed with vector reprogramming and qualifying in pilot experiments but is prone to a higher rate of guide‐barcode decoupling because several barcodes should be assigned to one cell. Approximately around the same time, Jaitin et al. developed a scCRISPR technique known as CRISP‐seq. However, they relied primarily on MARS‐seq, a fluorescence‐activated cell sorting (FACS)‐based scRNA‐seq technology.^[^ ^6^, ^12^ ^]^ Its inherent FACS‐based single‐cell sorting makes it simple to identify cell subtypes, making it ideal for analyzing isolated organisms. MARS‐seq is marginally inferior to microfluidics in terms of cell throughput due to the use of well plates instead of droplets as cell compartments (Table 1). Xie et al. also developed a technique known as Mosaic‐seq. Similarly, it incorporates a 12‐nt random barcode at the 3″end of the open reading frame of the backbone to accommodate the 3″‐biased sequencing platform Drop‐seq. To explore enhancer functions on a large scale, cells were disturbed at high MOI in Mosaic‐seq screens, and dCas9‐KRAB instead of Cas9 was employed to suppress enhancer functions.^[^ ^11b^ ^]^

The three methods mentioned here are the most primitive ones featured by barcoding systems. However, the efficiency of gene inhibition in these screens is restricted, with over one‐third of transduced cells exhibiting off‐target, in‐frame mutation, or non‐perturbed characteristics.^[^ ^13^ ^]^ These can result in meaningless data and resource waste.^[^ ^14^ ^]^ Tang et al.^[^ ^13^, ^15^ ^]^ proposed a double‐nicking technique to improve the efficiency of CRISPRko and conserve experimental resources. They constructed each plasmid with two sgRNA pairs targeting both ends of a single exon and nicked the targeted loci with Cas9n, which resulted in a robust large‐segment loss. Due to their design, DoNick‐seq, the on‐target accuracy increased to greater than 90 percent.

In addition to improving the knockout efficiency, efforts have been made to broaden the spectrum of competence. CRISPR knock‐in relies on homology‐directed repair and was recently developed for large‐scale gain‐of‐function tests on reprogrammed primary human T cells.^[^ ^16^ ^]^ Due to the poor transduction rate of primary cells, this approach, known as pooled knock‐in sequencing (PoKI‐seq), employs electroporation to transfer Cas9‐gRNA ribonucleoprotein (RNP) and exogenous DNAs. The exogenous DNA includes a synthetic gene flanked by T‐cell receptor (TCR) homology arms, where a unique barcode is embedded and can be detected by targeted sequencing.^[^ ^16^ ^]^ Additionally, small mismatches were positioned in the 3’ homology arm as a PCR blocker for quality control. To minimize template switching, the DNA library was not pooled until the electroporation step. As a ground‐breaking rich‐in‐content pooled screening method with CRISPR knock‐in, PoKI‐seq is expected to accelerate research on immune therapeutics.

As mentioned in Perturb‐seq, vectors containing multiple sgRNAs infer a “multiple guides per cell” paradigm for efficient investigation on complicated biological networks. However, it is not easy to scale to higher‐order combinations because the non‐uniformity of expression among aligned cassettes is hard to equalize.^[^ ^6a^ ^]^ In contrast, raising MOI is just a stopgap owing to its potential downsides such as cytotoxicity. Cas13d, a type VI CRISPR enzyme, has recently been shown to be easily programmable for RNA‐guided gene knockdown and combinatorial single‐cell screening. This assay is known as the Cas13 RNA Perturb‐seq (CaRPool‐seq) experiment.^[^ ^17^ ^]^ Structurally more compact than Cas9, Cas13d can be directed by crRNA itself to specifically hydrolyze RNAs and can naturally coordinate with the crRNA combinations. CaRPool‐seq, in contrast to Perturb‐seq, is driven by a single U6 promoter when targeting three sites. A fourth crRNA flanking the array (the barcode gRNA; bcgRNA) was modified with a barcode sandwiched by RT‐PCR adaptors. After expression, crRNA arrays will be Cas13d‐processed into separate crRNAs that can individually function. Thus, the expression of distinct crRNA is identical in CaRPool‐seq. The bcgRNA was released and detected by scRNA‐seq following RT‐PCR. In their paper, the authors benchmarked CaRPool‐seq with a 10× scRNA‐seq platform that is compatible with both transcriptome and surface‐protein read‐outs.

In vivo assays are attractive because their cellular environment is closer to real organic conditions. scRNA‐seq‐based CRISPR screening also includes dedicated designs for in vivo applications (**Table** **2**). The in vivo Perturb‐seq, proposed by Jin et al., partially followed the Perturb‐seq protocol but performed pooled perturbation in the prenatal murine forebrain instead of cell culture.^[^ ^18^ ^]^ To in situ perturb neocortical cells, they injected a dual guide RNA (dgRNA) library made of gRNA pairs targeting the same genes to improve knockout efficiency into the lateral ventricles of unborn mice. Then, postnatal mouse cells were extracted and utilized for scRNA‐seq. In vivo investigation of the immunological response of reprogrammed T cells to solid tumors was also shown to be viable using PoKI‐seq^[^ ^16^ ^]^ (Table 2).

**Table 2:** Summary of applications of the scCRISPR methods

Method	Mode	Subject	Effector	Effector delivery	Library delivery	Applications	Ref.
Perturb‐seq^a)^	In vitro	K562	dCas9‐KARB	Pre‐engineered	Lentivirus	Systematic analysis of UPR pathways and genome‐wide screens of relevant genes in a two‐tied strategy in K562 cells.	[6a]
	In vitro	K562	Cas9	Lentivirus	Lentivirus	Unraveling of a systemic transcriptional program in the K562 with TF knockout.	[6b]
CRISP‐seq^a)^	In vitro	Mouse BMDCs	Cas9	Pre‐engineered	Lentivirus	Revealing the effect of TFs on circuits rewiring of inflammatory and antiviral bio‐networks in Lipopolysaccharide (LPS) stimulated born marrow cells (BMCs).	[6c]
	In vivo	Mouse HPCs	Cas9	Pre‐engineered	Lentivirus	Analysis of the function of TFs in immune regulation circuits in murine splenic niche following LPS‐stimulation.	[6c]
Mosaic‐seq^a)^	In vitro	K562	dCas9‐KARB	Pre‐engineered	Lentivirus	Investigating super‐enhancer function within seven topologically associated domains (TADs).	[11b]
CROP‐seq^a)^	In vitro	Jurkat	Cas9	Pre‐engineered	Lentivirus	Screening for regulators and TFs regulating TCR activation in Jurkat cells.	[7a]
CROP‐seq‐2^a)^	In vitro	MCF10A cells	Cas9	Pre‐engineered	Lentivirus	Screening of tumor genes under doxorubicin.	[20]
crisprQTL mapping^a)^	In vitro	K562	dCas9‐KRAB	Pre‐engineered	Lentivirus	Enhancer‐gene pair screening within DNase I hypersensitive sites (DHS) in K562 cells.	[11a]
SLICE^a)^	In vitro	Human primary T cells	Cas9	Electroporation	Lentivirus	Assessing immune responses in primary T cells from cancer patients when negative or positive‐related genes are ablated.	[23]
CRISPRi‐I^3^N^a)^	In vitro	iPSCs	dCas9‐KRAB	TALEN	Lentivirus	Deepening understanding of the function of hit genes in iPSC and neuron survival.	[26]
In vivo Perturb‐seq^a)^	In vivo	Progenitor cells of the mouse forebrain	Cas9	Pre‐engineered	Lentivirus	Screening of ASD/ND risk genes in the mouse developing brain.	[18]
PoKI‐seq^a)^	In vivo	Human primary T cells; NSG mice bearing human melanoma	Cas9	Electroporation	Electroporation	Screening for T cell‐reprogramming constructs leads to improved tumor infiltration and cell killing rate.	[16]
TAP‐seq^a)^	In vitro	K562	dCas9‐KRAB	Pre‐engineered	Lentivirus	Mapping enhancer‐gene relations in loci on chromosome 8/11 in K562 cells.	[19]
SNV screening^a)^	In vitro	A375	CBE3	Transposon	Lentivirus	Investigating single nucleotide polymorphism (SNPs) inducing vemurafenib resistance in melanoma.	[21]
STING‐seq^a)^	In vitro	K562	dCas9‐KRAB	Lentivirus	Lentivirus	Defining regulatory networks of cis‐regulatory elements inside of blood traits GWAS loci.	[53a]
scCRISPRa screening^a)^	In vitro	Mouse embryonic stem cells	dCas9‐VP64	Lentivirus	Lentivirus	Identifying molecular signature of zygotic genomic activation (ZGA) and factors promoting ZGA‐like response.	[54]
Direct‐capture Perturb‐seq^a)^	In vitro	iPSCs, K562	Cas9, dCas9‐KRAB, dCas9‐SunTag/scFV‐VP64	Lentivirus	Lentivirus	Dissection of epistatic effects between DNA repair and cholesterol biogenesis.	[5]
Direct‐seq^a)^	In vitro	Jurkat, K562	Cas9, dCas‐MPH‐VPR	Lentivirus	Lentivirus	Validation of its performance on combinatorial perturbation.	[7b]
Scifi‐RNA‐seq^a)^	In vitro	Jurkat	Cas9	Lentivirus	Lentivirus	Genetic screen studying vital regulators of TCR activation.	[22b]
sc‐Tiling^a)^	In vitro	MLL‐AF9 transduced leukemic cells from mouse	Cas9	Pre‐engineered	Lentivirus	Characterization of the function of *DOT1L* coding region at sub‐gene resolution using trajectory analysis.	[27]
Transcript splicing screening^a)^	In vitro	HEK293T, Jurkat	Cas9	Pre‐engineered	Plasmid/electroporation/lentivirus	Exploring relationships between *RACK1* mRNA isoforms with splicing sites and PTPRC mRNA with splicing factors.	[28]
Genome‐scale Perturb‐seq^a)^	In vitro	K562, RPE1	dCas9‐KRAB (derived from *ZIM3* or *KOX1*)	Lentivirus	Lentivirus	Dissecting relationships between genes related to gene translation and ribosome biogenesis; clarifying mechanisms of aneuploidy.	[80]
DoNick‐seq^a)^	In vitro	HEK293T, HIEC	Cas9n	Plasmid/ lentivirus	Plasmid/ lentivirus	Analyzing bio‐effects of mTORC1 regulators under amino acids depletion in cell lines.	[13]
Expression CROP‐seq^a)^	In vitro	HL60/S4	Cas9	Lentivirus	Lentivirus	Assessing the influence of SNP within credible intervals to gene expression in myeloid cells.	[107]
CRISPRa Perturb‐seq	In vitro	Primary human T cells	dCas9‐VPH	Lentivirus	Lentivirus	Understanding cytokine‐dependent mechanisms of T cell regulation under TCR stimuli.	[108]
Perturb‐ATAC^b)^	In vitro	Primary human keratinocytes, B lymphoblasts	dCas9‐KRAB, Cas9	Lentivirus/ pre‐engineered	Lentivirus	Comparing the effects of 12 trans‐factors on the chromatin landscape of B cells, analyzing epistatic relation between factors.	[29]
Spear‐ATAC^b)^	In vitro	K562, GM12878, MCF7	dCas9‐KRAB	Pre‐engineered	Lentivirus	Revealing the temporal dynamics of epigenetic regulation in cancer cells and the relationships between TF binding patterns.	[30]
CRISPR‐sciATAC^b)^	In vitro	NIH‐3T3, K562	Cas9	Plasmid	Lentivirus	A pooled screen of chromatin modifiers and remodeling complexes with chromatin accessibility read‐out.	[55]
Pro‐Codes^c)^	In vitro, in vivo	293T, THP1, Jurkat, 4T1, Rag^‐/‐^ /BALB/c Mice, BALB/c Mice	Cas9	Lentivirus	Lentivirus	Revealing the mechanisms of cancer resistance to antigen‐specific T‐cell killing.	[10c]
Perturb‐map^c)^	In vitro, in vivo	293T, KP, 4T1, BALB/cJ mice, C57BL/6J mice	Cas9	Lentivirus/ pre‐engineered	Lentivirus	Tgfbr2 deficiency boosted the inhibitory effects of TGF*β* on the TME in lung cancer.	[39]
ECCITE‐seq^c)^	In vitro	Sez4, MyLa, PBMC, NIH‐3T3	Cas9	Plasmid	Lentivirus	Profiling of the five modalities followed by genetic perturbations in three cell lines.	[42]
Perturb‐CITE‐seq^c)^	In vitro	Melanoma cell, TIL	Cas9	Lentivirus	Lentivirus	Researching mechanisms of resistance in melanoma cells and TILs.	[25]
CaRPool‐seq^c)^	In vitro	THP‐1	Cas13d	Pre‐engineered	Lentivirus	Investigating regulator interactions in leukemic cell differentiation.	[17]
Imaging‐based CRISPR screen^d)^	In vitro	U‐2 OS cells	Cas9	Pre‐engineered	Lentivirus	Revealing regulators for nuclear RNA localization.	[64]
Optical pooled screen^d)^	In vitro	HeLa	Cas9	Pre‐engineered	Lentivirus	Identifying regulators of NF‐*κ*B activation.	[44b]
CRaft‐ID^d)^	In vitro	HEK293T	Cas9	Lentivirus	Lentivirus	Identifying and validating RNA‐binding proteins that contribute to stress granule formation.	[48]
MIC‐Drop^d)^	In vivo	Zebrafish embryo	Cas9	Nanodrop injection	Nanodrop injection	Genotype–phenotype relationships probing in zebrafish on a large scale.	[50]
Raft‐seq^d)^	In vitro	U2OS	Cas9	Lentivirus	Lentivirus	Mapping mitochondrial distribution abnormality to MFN2 variants.	[49]

^a)^ Indicates scCRISPR with RNA‐seq;

^b)^ Indicates scCRISPR with ATAC‐seq;

^c)^ Indicates scCRISPR with proteome probing;

^d)^ Indicates imaging‐based scCRISPR. “Pre‐engineered” denotes that the model is engineered with the CRISPR effector gene before the experiment by unknown approaches; “TALEN” denotes that the effector is engineered using TALEN techniques; “in vivo” denotes that cells are perturbed in situ within animal or zygote, or are perturbed ex vivo, then transplanted into animal subjects; “in vitro” indicates that the entire experiment is conducted in cell culture.

#### 2.1.2 Polyadenylated sgRNA

Although the combination of single‐cell barcoding and CRISPR editing is effective, its scalability is limited by the growing number of hypotheses requiring verification as the gRNA library grows.^[^ ^19^ ^]^ By comparison, the design of CROP‐seq allows direct detection of sgRNA spacers with scRNA‐seq. The CROP‐seq vector includes a sgRNA cassette in the 3′ long terminal repeat (LTR) of the lentiviral genome, downstream of the reporters and allowing their co‐transcription.^[^ ^7a^ ^]^ Since the reporters are driven by EF‐1*α*, a Pol II‐related promoter, the downstream sgRNA is polyadenylated and enables direct determination by poly(dT)‐based scRNA‐seq (Figure 3B). During transduction, duplication of sgRNA into the 5′‐LTR preserves its function. Thus, CROP‐seq significantly minimized identity mismatches. However, it has a slightly lower sgRNA recovery rate, which can be ameliorated by hemi‐nested PCR.^[^ ^20^ ^]^

The recalling strategy of CROP‐seq has been used as a pivot for other scCRISPR techniques (Table 1). One group modified CROP‐seq in a CRISPRi screen to simulate the expression quantitative trait loci and examined enhancer functions using their framework.^[^ ^11a^ ^]^ Their methodology, called crisprQTL mapping, is characterized by an extremely high MOI (28 sgRNAs per cell on average). In another study, CBE3, which can induce random C‐G to T‐A transversions in the activity window in protospacers, was employed to screen for single nucleotide variations (SNVs) that induce drug resistance.^[^ ^21^ ^]^ This method, referred to as “SNV screening” hereafter, is the only one that permits screening for SNVs (Table 1).

When it is necessary to measure low expression genes or subtle impacts, conventional scRNA‐seq with whole‐transcriptome analysis usually underperforms. Therefore, Schraivogel et al. recommended targeted Perturb‐seq (TAP‐seq). In addition to the PCR adapter for non‐specific cDNA amplification, TAP‐seq incorporates two specifically designed semi‐nested PCR primers to enrich gRNA transcripts and target genes.^[^ ^19^ ^]^ They optimized the design of primers to minimize off‐target effects and primer dimers. TAP‐seq outperformed Perturb‐seq with a significantly higher signal‐to‐noise ratio when using arranged primers.^[^ ^19^ ^]^ In other instances, an enormous number of cells is required to perform genome‐wide screens. Datlinger et al. developed a microfluidics‐based platform capable of phenotyping millions of cells in a single experiment to maximize cell throughput and reduce costs. This approach, known as scifi‐RNA‐seq, avoids the generation of wasted vacant droplets in microfluidic devices. They mirrored the combinatorial indexing strategy in SPLiT‐seq and adopted a single round of cell pre‐indexing before loading onto microfluidic devices.^[^ ^22^ ^]^ They revealed that a single droplet can be overloaded with up to ten nuclei or cells while remaining stable. It was discovered that scifi‐RNA‐seq yields a high‐quality sequencing library with fewer doublets, less noise, and 15‐fold higher throughput than SPLiT‐seq and 10× Chromium.^[^ ^19^ ^]^

Immortalized cells are used in the majority of scCRISPR studies. In these cells, some of the biological processes that are common in human cells do not occur, limiting their use in medical functional studies. To gain insights into the immunological activities of human primary T cells, Shifrut et al. combined CROP‐seq with electroporation delivery of CRISPR cargo to create a system called SLICE (sgRNA lentiviral infection combined with a Cas9 protein electroporation system).^[^ ^23^ ^]^ Arrayed RNP electroporation circumvents poor lentiviral transduction into human primary T cells and the time‐consuming gene expression phase, which is intolerable for post‐mitotic cells with their limited passage number.^[^ ^24^ ^]^ Electroporation delivery strategies have become a routine way to perturb primary cells, as illustrated by PoKI‐seq and Perturb‐CITE‐seq.^[^ ^16^, ^25^ ^]^ Neurons, another type of end cells that lacks human donors, were proven to be inducible from induced pluripotent stem cells (iPSCs) and used to perform CRISPR screens through Tian and colleagues' efforts. They generated a clonal dCas9‐KRAB expressing iPSC culture by TALEN knock‐in of dCas9 ‐KRAB cassette into the CLYBL safe harbor locus. Then, the iPSCs are transduced with a gRNA library. The subsequent NGN2‐induced glutamatergic neurons were referred to as the “isogenic, integrated, and inducible neurons” (i^3^N)^[^ ^26^ ^]^ (Table 1). With this CRISPRi‐i^3^N system, researchers can screen for complex phenotypes in easily available neurons.

#### 2.1.3 sgRNA with Capture Sequence

Given the success of polyadenylated sgRNA‐based scCRISPR approaches such as CROP‐seq, the complexity of genetic linkages necessitates the development of multiplexed perturbation methods. Except for using other effectors such as Cas13d, one of the solutions was found by Replogle et al. This cutting‐edge method, termed direct capture Perturb‐seq, exploits the CS spliced into optional positions of the sgRNA construct and complementary primers used for directly capturing sgRNA transcripts.^[^ ^5^ ^]^ Using this method, they repurposed the 10× Chromium 3′ (CS‐demanding) and 5’ (constant region as CS) scRNA‐seq kits to perform scCRISPR (Figure 3C). They demonstrated that their dgRNAs could synergize on single genes by utilizing dgRNA libraries. However, this approach is dependent on 10× Chromium‐exclusive CSs and probes and is incompatible with other sequencing platforms. Hence, Song et al. developed Direct‐seq, which followed similar concepts but utilized an A/G‐mixed CS that could anneal to poly(dT)‐based RT primers, making it platform‐independent.^[^ ^7b^ ^]^ They demonstrated that CSs can function at either of the three sites on the sgRNA construct, tetraloop, stem loop 2, or 3’ end, without affecting its activity. Specifically, with 3′ capture, Direct‐seq contains a transfer RNA sequence upstream of the spacer to serve as a reverse PCR handle. Both approaches utilizing guide‐targeted PCR were successful in CRISPRko/CRISPRa screening using dgRNA libraries.

Recent advances in functional genomics have incorporated direct capture Perturb‐seq (Table 1). Yang et al. integrated the 10× direct capture method with cell transcriptome atlas trajectory analysis and proposed their method (sc‐Tiling) as a tool for verifying functional domains at the sub‐gene level.^[^ ^27^ ^]^ They linked the transcriptome to the cryo‐electron microscopy structure of methyltransferase DOT1L, a druggable enzyme with many catalytic sites, and discovered novel domains that could not be identified using survival screens. Another study defined transcript isoforms in the absence of alternative splicing sites or splicing factors by using a combination of direct capture Perturb‐seq and single‐cell long‐read sequencing.^[^ ^28^ ^]^ The Oxford Nanopore was employed to capture whole transcripts, followed by 5’ short‐read sequencing to identify valid CBCs and GBCs. Combining short‐ and long‐read sequencing enables high‐throughput mRNA processing route mapping.

### 2.2 scCRISPR with ATAC‐seq

High‐content phenotyping of chromatin accessibility changes within perturbed cells provides new perspectives for exploring the association between CRISPR‐based gene perturbations and epigenetics. Several scCRISPR methodologies (Table 1) integrated with ATAC‐seq (Figure 2B) were developed to investigate epigenomic regulatory mechanisms and the interaction between regulators and chromatin.

Perturb‐ATAC emerged as a pioneer in single‐cell screening, with chromatin accessibility as a readout.^[^ ^29^ ^]^ After combining single immortalized B lymphoblasts expressing dCas9‐KRAB and sgRNA vectors with GBC in microchambers, they obtained cells with interference in TF SPI1, which is critical for B‐cell development. The cells were then purified by FACS, and the transduced cells were pooled and treated with Tn5 transposase. Following PCR amplification, the fragments of ATAC‐seq and reverse‐transcribed sgRNA were further amplified separately using CBC to generate libraries. The quantity of GBC and the quality of the ATAC‐seq fragment were evaluated, both of which illustrated low mutual interference and high accuracy to ensure the feasibility of Perturb‐ATAC. Finally, they matched GBC to the ATAC‐seq phenotype, and the expected results confirmed the technical pipeline. Subsequently, Perturb‐ATAC was expanded to larger‐scale scCRISPR. However, Perturb‐ATAC has several limitations, such as its throughput of single cells being limited to 96 cells per run, the generated ATAC‐seq fragment and cDNA were not separated in the first round of PCR, and its background noise generated by non‐targeted amplification is inevitably at a high level. Spear‐ATAC was thus developed.^[^ ^30^ ^]^ It works in the same way as Perturb‐ATAC, but it uses a specially designed sgRNA with pre‐integrated adapters and biotin‐tagged primers. These two modifications enabled preferential amplification and targeted enrichment of sgRNA fragments directly from genomic DNA with a minimized ATAC‐seq background noise. And Spear‐ATAC was applied to dissect the interaction between TFs and the dynamic effects of TF knockdown. Compared to Perturb‐ATAC, Spear‐ATAC achieves a substantial increase in cell throughput (between 35‐ and 100‐fold) and a significant cost reduction (20‐fold), offering a more practical alternative (Table 1).

In contrast to the modification of the sgRNA vector, Liscovitch‐Brauer and co‐workers performed two rounds of barcoding of ATAC‐seq fragments and sgRNAs, termed single‐cell combinatorial indexing ATAC‐seq (CRISPR‐sciATA).^[^ ^55^ ^]^ This technique demonstrates similar strength to Spear‐ATAC in terms of cell throughput and cost and provides a primary method for distinguishing gRNA in perturbed cells from uncaptured cells. However, this combinatorial barcoding strategy partly limits the detection of subtle changes in chromatin accessibility. CRISPR‐sciATAC should be combined with droplet‐based cell barcoding and more sophisticated algorithms to address these challenges.

### 2.3 scCRISPR with Proteome Probing

Proteome analysis reflects protein level from downstream of the central dogma,^[^ ^31^ ^]^ playing an irreplaceable role in high‐content screens,^[^ ^31^, ^32^ ^]^ especially in signal transduction, such as cell‐mediated immunity. More recently, using either cytometry or microfluidics,^[^ ^4^, ^33^ ^]^ two approaches for multiplexed single‐cell proteomics were developed.^[^ ^34^ ^]^ One utilizes tandem combinations of epitopes as barcodes that can be detected along with the cell proteome by mass cytometry by time‐of‐flight (CyTOF).^[^ ^10c^ ^]^ Extracellular proteins have also been probed by DNA‐conjugated antibodies, followed by microfluidics‐based single‐cell preparation and pooled sequencing.^[^ ^4^, ^35^ ^]^

In 2018, Wroblewska et al. developed the first highly multiplexed barcoding system enabling single‐cell genetic screening, termed Pro‐Codes, pushing the limits of low content and few parameters encountered in regular cytometry.^[^ ^10^, ^36^ ^]^ Pro‐Codes utilize ternary barcodes consisting of tandem linear epitopes that can bind to unique combinations of antibodies chelated with metal isotopes (Figure 3C). Furthermore, researchers fused the triplet with the truncated nerve growth factor receptor (dNGFR), a membrane‐bound protein, as a reporter antigen and scaffold to locate the barcode on the cell surface. Like Perturb‐seq, they assembled plasmids with U6‐initiated gRNA cassettes and EF‐1*α*‐initiated barcodes. After cell harvest and staining for dNGFR and barcodes, cells were atomized and analyzed with CyTOF (Figure 2C). In a pilot experiment, they demonstrated that co‐packaging of low‐homology vectors could significantly reduce template switching in pooled packaging.^[^ ^10^ ^c]^ In addition, the multiplexity of Pro‐Codes can be expanded by introducing additional epitopes or extra epitope positions for lineage tracing study.^[^ ^37^ ^]^ Pro‐Codes is extremely scalable at the library scale and cell throughput^[^ ^38^ ^]^ (Table 1). Recently, the same group repurposed and further expanded Pro‐Codes with immuno‐histological imaging and spatial transcriptomics to develop an evolved approach termed the Perturb‐map, they first designed nuclear‐localizing Pro‐Codes (nPC) with nuclear‐localizing mCherry (mCherry‐NLS) in place of dNGFR as a scaffold, in addition to the membrane‐bound Pro‐Codes (memPC) in the previous studies.^[^ ^39^ ^]^ Using a spatial proteomic technique called multiplexed immunohistochemistry consecutive staining on a single slide (MICSSS)^[^ ^40^ ^]^ and a multiplexed ion beam imager (MIBI), they demonstrated nPC as a better cell tag for microscopic observation and discrimination. In addition, they found that mixing two Pro‐Codes might result in the multiplication of the barcode number (over 3000 barcodes with 8 epitopes). Using nPC, MICSSS, and MIBI, the authors identified key genes that impact both tumor growth and histological features.^[^ ^39^ ^]^ Furthermore, the tissue slices were re‐stained with antibodies against lymphocyte antigens to investigate the tumor microenvironment (TME). Finally, they paired Perturb‐map with the10× Visium platform to recognize molecular markers.^[^ ^39^ ^]^ In short, by merging spatial transcriptomics, multiplexed imaging, and Pro‐Codes, Perturb‐map provides a versatile method for measuring genetic relationships and perturbation effects on the TME.

However, Pro‐Codes‐based approaches are restricted in terms of devices and protein‐based detection strategies and can detect fewer than 40 proteins simultaneously. Pro‐Codes screening, therefore, can only be utilized for gene screening of specific phenotypes and is far less accessible for genome‐wide screening than scCRISPR approaches based on DNA barcodes. Therefore, researchers look for protein‐based barcode sequencing methods, and CITE‐seq provides an attractive solution. CITE‐seq utilizes oligonucleotides (oligos) including PCR primers, antibody barcodes, and oligo‐dT primers to label antibodies.^[^ ^4^ ^b]^ After lysed the cells and indexing the antibody‐derived tags and sgRNAs with CBCs, the sequencing library is produced by RT and amplification. Cell Hashing, like CITE‐seq, adds sample barcodes to cells by tagging antibodies to ubiquitously expressed surface proteins, enabling multiplexing various samples and decreasing costs.^[^ ^41^ ^]^ These two approaches allow for multiplexing and doublet detection and are frequently employed in scCRISPR protein probing. Mimitou et al. created ECCITE‐seq to jointly capture transcriptome, TCR, surface protein, hashtag, and sgRNA.^[^ ^42^ ^]^ This data was then separated into three categories. First, the template switch oligo (TSO) carried by 10× Genomics 5P gem beads was used to add CBCs to the transcripts to detect the transcriptome and TCR during RT. The RNA fragments were then amplified to generate the first library. Then, CITE‐seq and Cell Hashing were used to acquire surface protein tags and hashing tags, and the same TSO‐based barcoding strategy was utilized to generate the library. To capture sgRNA, reverse‐transcription primers were designed specifically for targeted amplification. ECCITE‐seq decreases the number of required cells by combining different sequencing techniques. In addition, the technology is modular, allowing researchers to choose the optimal combinations for their research. Moreover, Fanngieh et al. developed Perturb‐CITE‐seq, a combination of Perturb‐seq and CITE‐seq. This method permits the simultaneous detection of sgRNAs, transcripts, and surface proteins, and it is consistent with previously described gRNA detection methods. In addition, they designed a computational framework utilizing dial‐out PCR data and a linear model with elastic network regularization. This framework provides a modular study of the impact of perturbations on RNAs and proteins by inferring important covariates in scCRISPR.

### 2.4 Imaging‐Based scCRISPR

scRNA‐seq, scATAC‐seq, and protein probing techniques have significantly increased screening efficacy in terms of genotype‐to‐phenotype mapping and decoding regulatory networks.^[^ ^43^ ^]^ However, these methods cannot determine certain phenotypes, such as cellular dynamics and morphology, mainly restrained by cell lysis before loading onto single‐cell platforms.^[^ ^44^ ^]^ On the other hand, microscopy has unparalleled power in the spatiotemporal monitoring of single cells but has not been well integrated with pooled screening. In recent years, the integration of pooled screens and microscopic imaging has emerged to address these limitations (Table 1).^[^ ^45^ ^]^ The imaging‐based methods were classified in the sgRNA identifying mechanism (Figure 2D), some of which captured optical barcodes by in situ sequencing (ISS), while others attached perturbation identity to a raft‐based physical separator (Table 2).

Owing to technical specificity, imaging‐based methods need to capture perturbation identity together with optical information. In Feldman et al. developed method, after snap‐shot of pooled cells, they used padlock‐based ISS to sequence either barcodes or sgRNAs (depending on what plasmid is used). Briefly, they conducted in situ RT, padlock extension/linkage, enzymatic amplification, and rolling circle amplification.^[^ ^44^, ^46^ ^]^ Such direct ISS methods add a spatiotemporal dimension to the analysis of gene knockouts and enable higher imaging throughput compared to arrayed assays. In addition, imaging‐based scCRISPR can be achieved by multiplexed error‐robust fluorescent in situ hybridization (MERFISH). However, this approach cannot directly detect multiplexed sgRNAs, in which each MERFISH barcode digit needs a long oligonucleotide to match. In the method developed by Wang et al., the sgRNA was aligned upstream to the barcode in the 3’ LTR, and downstream to the reporter gene, which mimics a CROP‐seq vector design but utilizes a barcode. After imaging, cells were subjected to rounds of hybridization of MERFISH probes with barcodes to identify perturbations to cells fixed in situ, and the same method was used to localize nuclear RNA and lncRNA and to identify the factors regulating RNA localization. MERFISH‐based approaches are more sensitive than direct ISS methods and are the only approach available for phenotyping bacteria.^[^ ^47^ ^]^ Although researchers have optimized vectors for long barcodes in MERFISH, these methods still have a high barcode‐sgRNA mismatching rate and require greater imaging magnification and bespoke library cloning methods.^[^ ^44a^ ^]^

Aside from fluorescent sequencing, there are single‐cell screening methods based on physical separation. CRaft‐ID,^[^ ^48^ ^]^ for example, separates perturbed cells using microrafts and then uses automatic high‐content confocal imaging of single microrafts to determine specific phenotypes. Compare to ISS and ISH, raft isolation is devoid of chemical processing and avoids limitations concerning chemical reagents. However, the manual isolation of microrafts reduces the efficiency of screening on the other hand,^[^ ^44a^ ^]^ and its resolution is limited at the colony level. Similarly, Raft‐seq employs raft isolation to separate single cells to be imaged and genotyped, but it allows for live cell phenotyping at single‐cell resolution and expands the imaging scope by raft design. Raft‐seq and CRaft‐ID both permits the collection of fixed or live cells, but those methods need cDNA preamplification for the least amount of material and one‐by‐one sequencing.^[^ ^49^ ^]^ Furthermore, Raft‐seq does not utilize any existing sgRNA detecting strategy but sequences the target gene indels as perturbation markers. Given that the authors merely investigate a single gene in their research, we recommend using the sgRNA identifier (barcode or sgRNA itself) in parallel with genes investigation.

A recent study developed the MIC‐Drop platform, which employs a single needle to continuously inject microdroplets into thousands of zebrafish embryos, snapping abnormal phenotypes at the macroscopic level.^[^ ^50^ ^]^ They used a QX‐200 droplet maker to manufacture nanoliter beads with four sgRNAs targeting a single gene to introduce robust allelic gene knockout. Droplets were combined and injected into hundreds of embryos, and large numbers of recipients were observed in pore plates, where the appearance and activity were used to determine phenotypes. Although this study did not profile single cells at the transcriptome level, this is a theoretical possibility that is expected to occur soon.

## 3 Biological Applications

**Figure 4:**
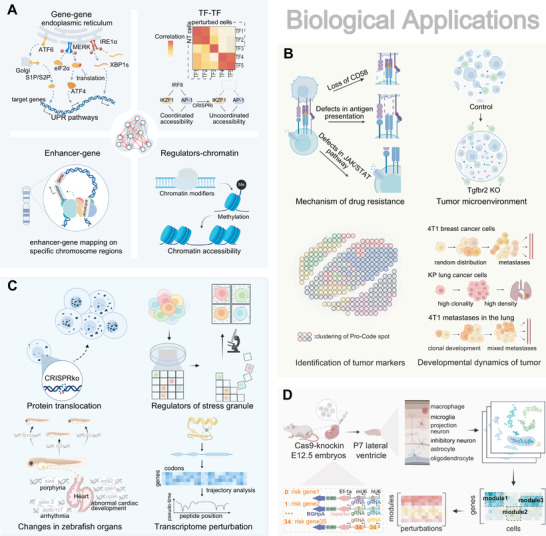
Biological applications of scCRISPR. A) Dissecting molecular regulatory mechanisms and interactions. scCRISPR is performed to uncover interactions between regulator genes and those genes involved in unfolded protein response (UPR). CRISPRi of IRF8 blocked TFs interaction, preventing AP‐1 factor activity from being coordinated with that of IKZF1. TAP‐seq was developed to map 1778 enhancers to corresponding genes. In CRISPR‐sciATAC, the effect of distinct perturbations of chromatin modifiers and remodelers on chromatin accessibility was demonstrated. B) scCRISPR offers a profound understanding of tumor biology. scCRISPR technologies are promising and potent tools for investigating tumor drug resistance mechanisms via gene perturbation at scale. Through genetic screening, the tumor microenvironment and markers can be characterized, and the metastasis dynamics of cancer cells can be monitored using Perturb‐map. C) Spatially resolved genotype‐to‐phenotype mapping. Imaging‐based scCRISPR connects perturbed genes to optical cellular phenotypes, including protein translocation, stress granule, the functional structure of a protein, and organic shapes in zebrafish. D) Investigating the pathogenesis of the development of the neural system. In vivo Perturb‐seq redefines gene functions and identifies the genetic mechanism of ASD/ND in the murine‐developing brain.

The versatility and robustness of scCRISPR approaches are demonstrated by their broad range of applications in various research topics, including spatially resolved phenotype‐to‐genotype mapping, genetic regulations, tumor biology, and pathogenesis in the developing neural system.^[^ ^51^ ^]^ The characteristic applications and critical experimental parameters of each scCRISPR method are summarized in Table 2, and the most representative applications of the cutting‐edge scCRISPR methods are illustrated in **Figure** **4**.

### 3.1 Dissecting Genetic Regulations

Complex relationships between genomic elements, including coding genes, transcription factors, chromatin regulators, enhancers, and other non‐coding elements, can be clarified using scCRISPR screening (Figure 4A). To discover the crosstalk between three UPR signal pathways, one example is Perturb‐seq using tri‐sgRNA (tgRNA) vectors to knock down three UPR sensor genes at low MOI.^[^ ^6^, ^52^ ^]^. In addition, a zoom‐in Perturb‐seq analysis performed after a genome‐wide fluorescent reporter screen revealed novel signaling patterns and the roles of specific genes in triggering UPR. Numerous enhancer‐gene pairings and epistatic interactions between tumor suppressor genes were discovered by scCRISPR when high MOI or rounds of transduction were used.^[^ ^11^, ^19^, ^53^ ^]^ The combination of scCRISPR with perturbations of TFs in myeloid dissects inflammatory and antiviral circuits concerning TCR stimulation in Jurkat cells^[^ ^7^, ^20^ ^]^ upon LPS stimulation.^[^ ^6c^ ^]^ A ZGA‐like transcriptional signature is elicited by TFs and epigenetic regulators, as discovered by activation‐based scCRISPR in mouse ESCs.^[^ ^54^ ^]^ Additionally, scCRISPR elucidated the mRNA splicing pattern of the receptors for activated C kinase 1 and protein tyrosine phosphatase receptor type C in combination with nanopore sequencing.^[^ ^28^ ^]^ Additionally, using scATAC‐seq, scCRISPR screenings revealed epigenetic landscape remodelers in human B lymphocytes and leukemia cells,^[^ ^29^, ^30^, ^55^ ^]^ and they verified non‐coding drivers of estrogen receptor‐dependent carcinogenic regulatory networks.^[^ ^56^ ^]^

In contrast to pooled screens, which merely statistically reveal which gene enhances cell viability, scCRISPR was created to reveal underlying mechanisms. A dgRNA scCRISPR screen presented a gene interaction (GI) map with cell status annotations and was able to construct a model to capture the rules regulating gene synthetic effects using a manifold learning algorithm.^[^ ^57^ ^]^ Importantly, the selective challenge affects how valuable scCRISPR screens are. The activities and interactions of host and viral genes during infection were therefore revealed by screening under conditions of cytomegalovirus and SARS‐CoV‐2.^[^ ^58^ ^]^ The identification of disease‐related transcriptional effects was also facilitated by screening differentiated precursor cells, including microglia and glutamatergic neurons generated from iPSCs.^[^ ^59^ ^]^ Ex vivo perturbation following in vivo cultivation, which can also be seen as a challenge, displayed a unique contribution to the perception of lymphocytic anti‐tumor immunological behavior.^[^ ^16^ ^]^

### 3.2 Paving New Roads to Tumor Biology

Thanks to the wide range of models available, scCRISPR has achieved exciting progress in fundamental tumor biology and therapeutics. For instance, Roth et al. screened for chimeric antigen receptors that enhanced T cell anti‐tumor functions under an immunosuppressive condition.^[^ ^16^ ^]^ Under vemurafenib selection, Jun et al. sought out all cytosine‐to‐thymine mutations inside the exons of three genes (MAP2K1, KRAS, and NRAS), and they discovered indels and transcriptome markers that contributed to drug resistance of melanoma^[^ ^21^ ^]^ (Figure 4B). Additionally, scCRISPR shed light on the mechanisms underlying the epithelial‐to‐mesenchymal transition,^[^ ^60^ ^]^ the interactions between oncogenic genes that cause epistasis,^[^ ^53^ ^]^ T cell exhaustion,^[^ ^61^ ^]^ differentiation blockade,^[^ ^62^ ^]^ the immune checkpoint regulation network,^[^ ^63^ ^]^ and the functions of druggable proteins.^[^ ^27^ ^]^

A range of intercellular events, such as tumor immune evasion, metastasis, and angiogenesis, can be vaguely understood by merely manipulating clonal cell lines in flasks and tubes because in vitro screening is typically restricted to intracellular inquiry. This can be addressed by conducting in vivo screens on immunodeficient mouse models that have received engraftments of human immune cells^[^ ^16^ ^]^ or on immunocompetent mice that have received tumor allotransplants.^[^ ^39^ ^]^ These screens could provide light on tumor morphology, histological characteristics, the recruitment of immune cells, and gene functions that influence the state of tumors and tumor‐infiltrating lymphocytes.^[^ ^16^, ^39^, ^61^ ^]^ Strictly speaking, in vivo scCRISPR is trail‐blazing but even far more immature than in vitro. Extending to cell types other than leukemia, myeloma, and melanoma, like pancreatic carcinoma and neuroendocrine tumor, which are still little known, would be desirable for both scCRISPR paradigms.

### 3.3 Spatially Resolved Phenotype‐to‐Genotype Mapping

Imaging or spatial transcriptomics (ST) technologies can generate spatial data consisting of multi‐dimensional information. With spatially resolved phenotyping and genotyping, scCRISPR has found precise and subtle correlations between phenotypes and genotypes. Imaging‐based screens, including those developed by Wang et al., Feldman et al., Wheeler et al., and Yenkin et al., were utilized to screen for genes regulating long non‐coding RNA location,^[^ ^64^ ^]^ p65 nuclear translocation,^[^ ^44^ ^b]^ stress granule abundance,^[^ ^48^ ^]^ and mitochondrial anomaly^[^ ^49^ ^]^ concerning neurodegenerative diseases (Figure 4C). All of these methods adopt machine learning models to estimate the cell state or fluorescence location, which indicates the importance of image recognition in massive phenotyping. Massively parallel screening in zebrafish embryos allowed for finding genes relating to porphyria, arrhythmia, and abnormal cardiac phenotypes.^[^ ^50^ ^]^ Because the size of embryos is large, they can be observed without a high‐powered microscope and fluorescent activation. Hence, it is possible to screen for time‐varying phenotypes. Another tiling application underscores the power of scCRISPR in dissecting domain interactions with the aid of 3D protein structure.^[^ ^27^ ^]^ Spatial proteomics has been combined with scCRISPR.^[^ ^10^ ^c]^ With fluorescent activation on tissue slices, it can be used to describe histological traits of lung tumorigenesis in vivo and has the potential to generalize to histological inspection on tumor or microorganisms invaded tissue. Finally, ST can associate spatial transcriptomic phenotypes with genotypes. Given the current used Visium technique can hardly reach a single‐cell resolution, the gene expression changes found were lesion‐ but not cell‐specific. To generalize ST to depict phenotypes at single‐cell resolution, future efforts are needed to enhance resolution power to the micron level.

### 3.4 Insights into the Neuropathological Mechanisms

Neurological diseases have a substantial genetic foundation in their pathophysiology, making etiology research and therapies challenging. Utilizing scCRISPR on neuron and neuroglial models has revealed the mechanisms of neurological disorders such as autism spectrum disorder (ASD),^[^ ^18^, ^65^ ^]^ neurodevelopmental delay (ND),^[^ ^18^, ^66^ ^]^ and Alzheimer's disease (AD).^[^ ^59a^ ^]^ In particular, Perturb‐seq performed on a dgRNA library targeting a collection of neuropathic risk genes in vivo identified genes involved in the prenatal development of the mouse brain as well as gene coregulation modules associated with five distinct subpopulations (Figure 4D). For instance, they discovered that the expression of Ank2 in Ndnf^+^ interneurons correlates substantially with interneuron differentiation and that the inhibition of Chd8 impairs oligodendrocyte differentiation during neocortex development. It is hoped that the in vivo Perturb‐seq platform can be used for other brain lumens and human organoids to aid in the analysis of secretory or interaction regulations. In contrast, although researches for human neuronal characteristics were restricted to iPSC‐derived progenitors, neurons, and microglia, they nonetheless yielded informative insights regarding neuropathogenesis. scCRISPR revealed gene modules on neural progenitor cells that either delay or promote neuron development.^[^ ^65^ ^]^ scCRISPR identified cell state regulators governing cell survival, phagocytosis, and inflammatory responses in neurons and microglia generated from iPSCs.^[^ ^26^, ^59^ ^]^ The model diversity of scCRISPR applied to neurological diseases is currently limited. The focus of future research will move to various cell types, resulting in a more comprehensive perturbation map of the entire brain.

## 4 Application‐Oriented and Programmable Guidelines for Individualized Demand

**Figure 5:**
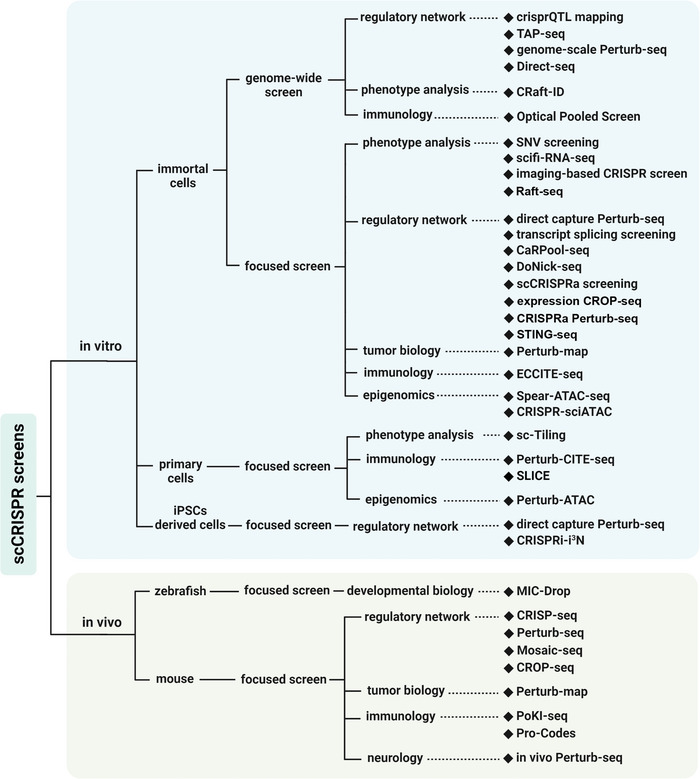
Decision diagram of appropriate scCRISPR methods for specialized research. All scCRISPR methods are categorized based on whether they support in vivo application, the experimental subject used in published studies, whether support genome‐wide screening and their potential biological application. Some methods may appear multiple times.

We developed a guideline for choosing the most appropriate scCRISPR methods for performing wet experiments based on the applications and technical features reported in their publications. First, scCRISPR methods diverge to specialize in either in vitro or in vivo applications (**Figure** **5**). In vitro experiments have several advantages over in vivo experiments, especially tentative experiments. In vitro experiments are simpler in design, more cost‐effective, and time‐saving because they do not require de novo cultivation and caring of experimental models. Moreover, owing to the reduced complexity, in vitro trials could have a higher chance of success. However, in vivo methods are better for confronting research topics of rare cell subsets and cell communication. Second, in vitro methods successful with immortal cell lines deviate from those with other cell types, including primary cells and neurons derived from iPSCs (Figure 5). Immortalized cells, including cancer cells and reprogrammed somatic cells, are widely used for proof‐of‐concept experiments because mature protocols for plasmid delivery are available and their passage numbers are almost unlimited.^[^ ^1^ ^b]^ In comparison, there remain unanswered questions regarding the efficient delivery of CRISPR cargo into certain cell types, such as suspension cells and primary cells.^[^ ^67^ ^]^ However, primary cells or iPSC‐derived neurons are irreplaceable when studying certain signaling pathways or phenotypes, such as neuronal morphology, owing to their unique transcriptional programs. Thirdly, we underlined genome‐wide screens that are presently confined to immortal cells (Figure 5). Although a focused screen is efficient for hit validation following a bulk screen, there should be genome‐scale methods allowing for mapping relations between genetic elements spread over the whole genome, or when a suitable bulk trait is inaccessible.^[^ ^1^, ^6^, ^11^ ^]^ Researchers may not have unveiled the maximum scale of their methods in their pioneering essays (Table 1).^[^ ^22^ ^b]^ Thus, it is meaningful for this review to provide a guideline to explore the optimal solution for scCRISPR screens. This guideline enables us to make a preliminary choice for the demand of research, such as the general pattern of interactions of specific gene regulators can be characterized with four methods selected by following “in vivo → immortal cells → focused screen → regulatory network.” And assuming you are not interested in mRNA isoforms but are concerned with combinatorial perturbation of at least three genes, CaRPool‐seq may be more suitable.

## 5 High‐Content Data Analysis

Conventional pooled CRISPR screening mainly relies on sgRNA frequency readout, which can be correlated with cell survival or FACS readout for perturbed cells. In contrast, scCRISPR screening with single‐cell sequencing or imaging readout, rich‐phenotype data, enables gaining insights into a complex regulatory network at high resolution and resolves the cellular heterogeneity of perturbations. The processing of complicated and abundant scCRISPR data remains challenging in computational tools and statistical algorithms. The analysis of scCRISPR data includes sgRNA identification and single‐cell sequencing/imaging data processing, which are typically processed separately and connected by CBCs or fluorescent protein markers. The identification and alignment of sgRNAs can be implemented by well‐document bioinformatic tools like MAGeCK,^[^ ^68^ ^]^ CERES,^[^ ^69^ ^]^ Bowtie,^[^ ^70^ ^]^ etc. The analysis for single‐cell sequencing data depends on the readout type, such as chromVAR^[^ ^71^ ^]^ for ATAC‐seq analysis, Seurat^[^ ^72^ ^]^ and Scanpy^[^ ^73^ ^]^ for transcriptome sequencing data. Analysis for single‐cell imaging data usually is performed with a custom processing script based on the experiments or carried out by high‐throughput image analytical software.^[^ ^74^ ^]^ The annotation of single‐cell readout with associated sgRNA is performed using bespoke scripts or bioinformatics tools such as Cell Ranger.^[^ ^75^ ^]^ Many analytical tools have been developed for quality control and normalization of single‐cell sequencing data in single‐cell communities, and FBA,^[^ ^76^ ^]^ Normalisr,^[^ ^77^ ^]^ and scMAGecK^[^ ^78^ ^]^ have been specifically designed for scCRISPR high‐content sequencing data to remove technical artifacts and correct batch effects among cells.

Technical noises from both CRISPR and single‐cell sequencing or imaging remain a challenge in the processing of scCRISPR data. The MUSIC,^[^ ^79^ ^]^ a topic modeling, and cell ranger^[^ ^75^ ^]^ can serve as more robust analytical methods for enhancing the signal‐to‐noise ratio of sgRNA efficiency and sparse single‐cell data. Due to the intricacy of high‐dimensional data readout in scCRISPR, it is difficult to examine thoroughly the sgRNA/perturbation effect on the state of single cells represented by rich‐phenotype. Several distance‐based algorithms in scRNA‐seq analysis, including energy distance,^[^ ^80^ ^]^ neighborhood‐based measures,^[^ ^81^ ^]^ and distances in learned autoencoder latent spaces,^[^ ^82^ ^]^ are used to assess the effect of CRISPR perturbations based on single‐cell sequencing data. In addition, the regularized linear models in MIMOSCA,^[^ ^6b^ ^]^ manifold learning in MELD,^[^ ^81a^ ^]^ and topic modeling in MUSIC^[^ ^79^ ^]^ provide quantification and estimation of the perturbation effect. Dissecting the heterogeneity among perturbed cells requires the identification of non‐perturbed cells with small sgRNA effects, hence, mixscape^[^ ^63^ ^]^ was created to eliminate confounding variation.

The abundant rich‐phenotype datasets generated by an increasing number of scCRISPR screens are highly valuable as data resources including multi‐modal phenotypes for driving the advancement of scCRISPR techniques and data mining. The well‐annotated metadata across a large number of scCRISPR datasets can be used as references for optimizing experimental parameters in scCRISPR screens. Manifold learning‐ and latent variable‐based models^[^ ^79^, ^81^ ^]^ can be applied to data mining in scCRISPR datasets to infer the perturbations effect. The annotated datasets from the collection of scCRISPR screens can contribute to genome‐wide association studies,^[^ ^83^ ^]^ and help validate the cell type‐specific GI in complex regulatory systems and the regulons predicted by gene co‐expression predicting tools like SCENIC.^[^ ^84^ ^]^ The development of bioinformatics tools for data mining to evaluate comprehensively the well‐curated multi‐modal scCRISPR datasets can facilitate the investigation of complex regulatory networks based on both epigenomics and transcriptomics.

## 6 Conclusions and Future Perspectives

Single‐cell CRISPR screening is paving the road toward reliable, high‐content massively parallel genetic screens. Researchers have developed tailor‐made sgRNA detection strategies covering different read‐outs (transcriptomes, proteomes, chromatin accessibility, and cell images). These developments have linked single‐cell phenotyping to CRISPR‐based genetic perturbations, rendering scCRISPR unprecedentedly powerful in genomic studies in various physiopathological models. We envision that progress in stimuli, read‐outs, sgRNA libraries, and models will continue to enhance the practicality and technical scalability of scCRISPR methods in the future.

Different from traditional pooled CRISPR screening with viability or reporter as read‐outs, scCRISPR enables single‐cell profiling of multi‐omic landscape and provides a deep understanding of regulatory mechanisms, and can characterize the cell heterogeneity in genetic perturbations to identify cell type‐specific responses to perturbations. And the experiment cycle of scCRISPR only depends on the response time of models to the stimuli and without cell competition.^[^ ^27^ ^]^ scCRISPR screens, where the whole perturbation library is delivered, activated, and finally extracted in a pool, avoid repetitive manipulation for every single perturbation, which significantly reduces batch effects, time, and economic costs. Unlike the one‐dimensional criteria used in guide‐ranking CRISPR screens, multi‐modal data from scCRISPR could be used to identify and validate hits with high fidelity. Annotating every single cell with sgRNAs in scCRISPR allows screening for gene pairs or elucidating genetic interactions with ease. Furthermore, these single‐cell datasets can be downsampled to subpopulations after unsupervised clustering, catering to personalized needs. However, the scCRISPR experiment is still unaffordable for most laboratories because of the high cost of single‐cell devices and reagents.^[^ ^11^ ^b]^ Limited to the number of single cells prepared with devices, each perturbation covers a small number of cells so that one perturbation can't be fully “exposed,”^[^ ^54^ ^]^ which results in the variation of sgRNA efficiency and low signal‐to‐noise ratio in readout data. Besides, preparing of single‐cell sample is quite difficult and requires harsh conditions for sample processing and preservation. It is more advisable to perform scCRISPR screens on commercialized single‐cell platforms such as 10× Chromium. The main obstacle faced by imaging‐based scCRISPR is that increasing the imaging area means more imaging time and economic costs. Processing high‐content scCRISPR readout data requires more complex informatics analytical tools and statistical skills than sgRNA frequency‐based pooled CRISPR screening, which indirectly limits the wider use of scCRISPR screening. scCRISPR applications will be substantially facilitated by the development of flexible and modular analytical tools. In addition, the multimodal scCRISPR phenotyping data can serve as a mineral‐rich resource for data mining to uncover novel regulatory mechanisms and enhance the accuracy of predictive models of genetic interactions.^[^ ^57^, ^82^, ^85^ ^]^ With technological advancement in single‐cell and the development of novel CRISPR systems engineered,^[2a,4c,^ ^2^, ^4^, ^86^ ^]^ the scCRISPR family could be enlarged and applied in a wider range of applications.^[^ ^87^ ^]^

Adding a spatial dimension to CRISPR‐based screening is very exciting.^[^ ^88^ ^]^ Recent advances in ST and proteomics have brought innovative insights into biological processes. The combination of CRISPR screening and spatially resolved phenotyping can investigate the spatial heterogeneity in a highly complex genetic regulatory system, which is expected to provide insights into neurodevelopment and neurodegeneration,^[^ ^89^ ^]^ interactions between lymphocyte and antigen‐presenting cells,^[^ ^90^ ^]^ pathological angiogenesis,^[^ ^91^ ^]^ and disease organoids.^[^ ^92^ ^]^ Currently, only the spatial scCRISPR based on barcoded protein locating has been developed to clarify tumor‐lymphocyte interactions,^[^ ^39^ ^]^ and RNA localization.^[^ ^64^ ^]^ And CRISPR screening with ST‐based phenotyping is limited by pixelated resolution and experimental complexity. These ST techniques that allow for single‐cell phenotyping^[^ ^93^ ^]^ can theoretically measure the sgRNAs and whole transcriptomes within a single cell by sequencing. The experimental complexity and low scalability of imaging‐based STs such as smFISH^[^ ^94^ ^]^ and MERFISH,^[^ ^95^ ^]^ also result in difficulties in integrating CRISPR screening.^[^ ^88^ ^]^ On the other hand, temporally resolved screening is yet limited to imaging‐based CRISPR screens,^[^ ^44^ ^b]^ or pseudo‐time trajectory generated from parallel cultures.^[^ ^21^, ^30^, ^56^, ^96^ ^]^ Recent advances in non‐invasive single‐cell transcriptomics (Live‐seq),^[^ ^97^ ^]^ might braze a trail. Metabolomics has intrinsic temporal properties and single‐cell dependency.^[^ ^98^ ^]^ To date, CRISPR screening with single‐cell metabolomics read‐out is still a blank. We anticipate that the isotope‐based barcoding system^[^ ^99^ ^]^ and a sub‐cellular pipetting approach^[^ ^97^ ^]^ may contribute to developing scCRISPR with metabolomics readout.

Off‐target effects are prevalent in CRISPR‐based editing processes. In single‐cell CRISPR knockdown screens, off‐target effects frequently induce DNA damage resulting in cell death, which not only decreases the cellular coverage of individual sgRNA but also introduces noise in single‐cell data, especially in essential gene screening. However, in other single‐cell CRISPR screens, such as CRISPR interference and CRISPR activation screens, off‐target effects have a lesser impact. In silico tools can be used to predict the target sequences with a low frequency of off‐target before screening,^[^ ^100^ ^]^ and optimal sgRNAs can be also selected from previously developed sgRNA libraries.^[^ ^101^ ^]^ Additionally, novel Cas variants such as ZIM3‐derived dCas9‐KRAB, and Cas13d could be utilized to reduce off‐targets in combinatorial screening.^[^ ^17^, ^102^ ^]^ And strategies that use dgRNA library and double nicking have also been proven to lessen off‐target effects.^[^ ^13^, ^18^, ^80^, ^102^ ^]^ It is recommended to substitute knockout with knockdown in case of DNA‐damage response, particularly when targeting short non‐coding elements.^[^ ^103^ ^]^ For genetic tiling and SNV screening, it is almost inevitable to utilize every target sequence with a valid protospacer adjacent motif (PAM), making these screens more susceptible to off‐target effects.^[^ ^21^, ^27^ ^]^ A recent discovery of a PAM‐free Cas9 variant holds promise for expanding the sgRNA library space and reducing off‐target in tiling screens.^[^ ^104^ ^]^

Existing scCRISPR screens have been performed mainly in vitro, whereas in vivo screens have a higher acceptance but are more technically challenging. Some in vivo studies have transplanted perturbed cells into living subjects and have given more predictable results.^[^ ^6^, ^10^, ^39^ ^]^ Other methods of in situ transduction within living tissues or zygotes^[^ ^18^, ^50^ ^]^ can track cell dynamics during tissue development and collect different cell subtypes at once (Table 2). However, in vivo assays are limited by their dependence on cell type, cytotoxic effects, or transduction failure, partly due to the immunogenicity of viral vectors.^[^ ^105^ ^]^ Recent advances have been made in the delivery systems of CRISPR effectors in vivo using modified viral and non‐viral approaches.^[^ ^86^, ^106^ ^]^ In vivo, scCRISPR screening for functional genomics can be applied to different model organisms such as yeast, *E. Coli*, and *Oryza sativa L*. Methods that allow in vivo scCRISPR screening will hopefully lead to paradigm‐shifting advances in research areas such as bioengineering, infectious disease control, and transgenic agriculture.^[^ ^58a^ ^]^

## Conflict of Interest

The authors declare no conflict of interest.

## References

[1] a) L. Przybyla , L. A. Gilbert , Nat. Rev. Genet. 2022, 23, 89; 34545248 10.1038/s41576-021-00409-w

[2] a) A. V. Anzalone , P. B. Randolph , J. R. Davis , A. A. Sousa , L. W. Koblan , J. M. Levy , P. J. Chen , C. Wilson , G. A. Newby , A. Raguram , D. R. Liu , Nature 2019, 576, 149; 31634902 10.1038/s41586-019-1711-4 PMC6907074

[3] J. G. Doench , Nat. Rev. Genet. 2018, 19, 67. 29199283 10.1038/nrg.2017.97

[4] a) J. D. Buenrostro , P. G. Giresi , L. C. Zaba , H. Y. Chang , W. J. Greenleaf , Nat. Methods 2013, 10, 1213; 24097267 10.1038/nmeth.2688 PMC3959825

[5] J. M. Replogle , T. M. Norman , A. Xu , J. A. Hussmann , J. Chen , J. Z. Cogan , E. J. Meer , J. M. Terry , D. P. Riordan , N. Srinivas , I. T. Fiddes , J. G. Arthur , L. J. Alvarado , K. A. Pfeiffer , T. S. Mikkelsen , J. S. Weissman , B. Adamson , Nat. Biotechnol. 2020, 38, 954. 32231336 10.1038/s41587-020-0470-y PMC7416462

[6] a) B. Adamson , T. M. Norman , M. Jost , M. Y. Cho , J. K. Nunez , Y. Chen , J. E. Villalta , L. A. Gilbert , M. A. Horlbeck , M. Y. Hein , R. A. Pak , A. N. Gray , C. A. Gross , A. Dixit , O. Parnas , A. Regev , J. S. Weissman , Cell 2016, 167, 1867; 27984733 10.1016/j.cell.2016.11.048 PMC5315571

[7] a) P. Datlinger , A. F. Rendeiro , C. Schmidl , T. Krausgruber , P. Traxler , J. Klughammer , L. C. Schuster , A. Kuchler , D. Alpar , C. Bock , Nat. Methods 2017, 14, 297; 28099430 10.1038/nmeth.4177 PMC5334791

[8] T. Baslan , J. Hicks , Nat. Rev. Cancer 2017, 17, 557. 28835719 10.1038/nrc.2017.58

[9] D. Feldman , A. Singh , A. J. Garrity , P. C. Blainey , bioRxiv 2018, 262121.

[10] a) B. Adamson , T. M. Norman , M. Jost , J. S. Weissman , bioRxiv 2018, 298349;

[11] a) M. Gasperini , A. J. Hill , J. L. McFaline‐Figueroa , B. Martin , S. Kim , M. D. Zhang , D. Jackson , A. Leith , J. Schreiber , W. S. Noble , C. Trapnell , N. Ahituv , J. Shendure , Cell 2019, 176, 377; 30612741 10.1016/j.cell.2018.11.029 PMC6690346

[12] D. A. Jaitin , E. Kenigsberg , H. Keren‐Shaul , N. Elefant , F. Paul , I. Zaretsky , A. Mildner , N. Cohen , S. Jung , A. Tanay , I. Amit , Science 2014, 343, 776. 24531970 10.1126/science.1247651 PMC4412462

[13] Y. Tang , S. Liao , G. Liu , X. Xiong , H. Liu , F. Li , Z. Tan , X. Kong , Y. Yin , B. Tan , Cell Biol. Toxicol. 2022, 38, 43. 33586084 10.1007/s10565-021-09586-0

[14] Y. Fu , J. A. Foden , C. Khayter , M. L. Maeder , D. Reyon , J. K. Joung , J. D. Sander , Nat. Biotechnol. 2013, 31, 822. 23792628 10.1038/nbt.2623 PMC3773023

[15] F. A. Ran , P. D. Hsu , J. Wright , V. Agarwala , D. A. Scott , F. Zhang , Nat. Protoc. 2013, 8, 2281. 24157548 10.1038/nprot.2013.143 PMC3969860

[16] T. L. Roth , P. J. Li , F. Blaeschke , J. F. Nies , R. Apathy , C. Mowery , R. Yu , M. L. T. Nguyen , Y. Lee , A. Truong , J. Hiatt , D. Wu , D. N. Nguyen , D. Goodman , J. A. Bluestone , C. J. Ye , K. Roybal , E. Shifrut , A. Marson , Cell 2020, 181, 728. 32302591 10.1016/j.cell.2020.03.039 PMC7219528

[17] H.‐H. Wessels , A. Méndez‐Mancilla , E. Papalexi , W. M. Mauck , L. Lu , J. A. Morris , E. Mimitou , P. Smibert , N. E. Sanjana , R. Satija , *bioRxiv* 2022, 2022.02.02.478894. 10.1038/s41592-022-01705-x PMC10030154 36550277

[18] X. Jin , S. K. Simmons , A. Guo , A. S. Shetty , M. Ko , L. Nguyen , V. Jokhi , E. Robinson , P. Oyler , N. Curry , G. Deangeli , S. Lodato , J. Z. Levin , A. Regev , F. Zhang , P. Arlotta , Science 2020, 370, eaaz6063. 33243861 10.1126/science.aaz6063 PMC7985844

[19] D. Schraivogel , A. R. Gschwind , J. H. Milbank , D. R. Leonce , P. Jakob , L. Mathur , J. O. Korbel , C. A. Merten , L. Velten , L. M. Steinmetz , Nat. Methods 2020, 17, 629. 32483332 10.1038/s41592-020-0837-5 PMC7610614

[20] A. J. Hill , J. L. McFaline‐Figueroa , L. M. Starita , M. J. Gasperini , K. A. Matreyek , J. Packer , D. Jackson , J. Shendure , C. Trapnell , Nat. Methods 2018, 15, 271. 29457792 10.1038/nmeth.4604 PMC5882576

[21] S. Jun , H. Lim , H. Chun , J. H. Lee , D. Bang , Commun. Biol. 2020, 3, 154. 32242071 10.1038/s42003-020-0888-2 PMC7118117

[22] a) A. B. Rosenberg , C. M. Roco , R. A. Muscat , A. Kuchina , P. Sample , Z. Yao , L. T. Graybuck , D. J. Peeler , S. Mukherjee , W. Chen , S. H. Pun , D. L. Sellers , B. Tasic , G. Seelig , Science 2018, 360, 176; 29545511 10.1126/science.aam8999 PMC7643870

[23] E. Shifrut , J. Carnevale , V. Tobin , T. L. Roth , J. M. Woo , C. T. Bui , P. J. Li , M. E. Diolaiti , A. Ashworth , A. Marson , Cell 2018, 175, 1958. 30449619 10.1016/j.cell.2018.10.024 PMC6689405

[24] a) A. Seki , S. Rutz , J. Exp. Med. 2018, 215, 985; 29436394 10.1084/jem.20171626 PMC5839763

[25] C. J. Frangieh , J. C. Melms , P. I. Thakore , K. R. Geiger‐Schuller , P. Ho , A. M. Luoma , B. Cleary , L. Jerby‐Arnon , S. Malu , M. S. Cuoco , M. Zhao , C. R. Ager , M. Rogava , L. Hovey , A. Rotem , C. Bernatchez , K. W. Wucherpfennig , B. E. Johnson , O. Rozenblatt‐Rosen , D. Schadendorf , A. Regev , B. Izar , Nat. Genet. 2021, 53, 332. 33649592 10.1038/s41588-021-00779-1 PMC8376399

[26] R. Tian , M. A. Gachechiladze , C. H. Ludwig , M. T. Laurie , J. Y. Hong , D. Nathaniel , A. V. Prabhu , M. S. Fernandopulle , R. Patel , M. Abshari , M. E. Ward , M. Kampmann , Neuron 2019, 104, 239. 31422865 10.1016/j.neuron.2019.07.014 PMC6813890

[27] L. Yang , A. K. N. Chan , K. Miyashita , C. D. Delaney , X. Wang , H. Li , S. P. Pokharel , S. Li , M. Li , X. Xu , W. Lu , Q. Liu , N. Mattson , K. Y. Chen , J. Wang , Y. C. Yuan , D. Horne , S. T. Rosen , Y. Soto‐Feliciano , Z. Feng , T. Hoshii , G. Xiao , M. Muschen , J. Chen , S. A. Armstrong , C. W. Chen , Nat. Commun. 2021, 12, 4063. 34210975 10.1038/s41467-021-24324-0 PMC8249386

[28] H. S. Kim , S. M. Grimes , A. C. Hooker , B. T. Lau , H. P. Ji , Genome Biol. 2021, 22, 331. 34872615 10.1186/s13059-021-02554-1 PMC8647366

[29] A. J. Rubin , K. R. Parker , A. T. Satpathy , Y. Qi , B. Wu , A. J. Ong , M. R. Mumbach , A. L. Ji , D. S. Kim , S. W. Cho , B. J. Zarnegar , W. J. Greenleaf , H. Y. Chang , P. A. Khavari , Cell 2019, 176, 361. 30580963 10.1016/j.cell.2018.11.022 PMC6329648

[30] S. E. Pierce , J. M. Granja , W. J. Greenleaf , Nat. Commun. 2021, 12, 2969. 34016988 10.1038/s41467-021-23213-w PMC8137922

[31] a) B. Schwanhausser , D. Busse , N. Li , G. Dittmar , J. Schuchhardt , J. Wolf , W. Chen , M. Selbach , Nature 2011, 473, 337; 21593866 10.1038/nature10098

[32] H. Gong , X. Wang , B. Liu , S. Boutet , I. Holcomb , G. Dakshinamoorthy , A. Ooi , C. Sanada , G. Sun , R. Ramakrishnan , Sci. Rep. 2017, 7, 2776. 28584233 10.1038/s41598-017-03057-5 PMC5459813

[33] a) E. Z. Macosko , A. Basu , R. Satija , J. Nemesh , K. Shekhar , M. Goldman , I. Tirosh , A. R. Bialas , N. Kamitaki , E. M. Martersteck , J. J. Trombetta , D. A. Weitz , J. R. Sanes , A. K. Shalek , A. Regev , S. A. McCarroll , Cell 2015, 161, 1202; 26000488 10.1016/j.cell.2015.05.002 PMC4481139

[34] L. F. Vistain , S. Tay , Trends Biochem. Sci. 2021, 46, 661. 33653632 10.1016/j.tibs.2021.01.013 PMC11697639

[35] a) B. Hwang , D. S. Lee , W. Tamaki , Y. Sun , A. Ogorodnikov , G. C. Hartoularos , A. Winters , B. Z. Yeung , K. L. Nazor , Y. S. Song , E. D. Chow , M. H. Spitzer , C. J. Ye , Nat. Methods 2021, 18, 903; 34354295 10.1038/s41592-021-01222-3 PMC8643207

[36] O. Shalem , N. E. Sanjana , F. Zhang , Nat. Rev. Genet. 2015, 16, 299. 25854182 10.1038/nrg3899 PMC4503232

[37] S. C. Bendall , E. F. Simonds , P. Qiu , A. D. Amir el , P. O. Krutzik , R. Finck , R. V. Bruggner , R. Melamed , A. Trejo , O. I. Ornatsky , R. S. Balderas , S. K. Plevritis , K. Sachs , D. Pe'er , S. D. Tanner , G. P. Nolan , Science 2011, 332, 687. 21551058 10.1126/science.1198704 PMC3273988

[38] G. Mullokandov , A. Baccarini , A. Ruzo , A. D. Jayaprakash , N. Tung , B. Israelow , M. J. Evans , R. Sachidanandam , B. D. Brown , Nat. Methods 2012, 9, 840. 22751203 10.1038/nmeth.2078 PMC3518396

[39] M. Dhainaut , S. A. Rose , G. Akturk , A. Wroblewska , S. R. Nielsen , E. S. Park , M. Buckup , V. Roudko , L. Pia , R. Sweeney , J. L.e Berichel , C. M. Wilk , A. Bektesevic , B. H. Lee , N. Bhardwaj , A. H. Rahman , A. Baccarini , S. Gnjatic , D. Pe'er , M. Merad , B. D. Brown , Cell 2022, 185, 1223. 35290801 10.1016/j.cell.2022.02.015 PMC8992964

[40] R. Remark , T. Merghoub , N. Grabe , G. Litjens , D. Damotte , J. D. Wolchok , M. Merad , S. Gnjatic , Sci. Immunol. 2016, 1, aaf6925. 28783673 10.1126/sciimmunol.aaf6925 PMC10152404

[41] M. Stoeckius , S. Zheng , B. Houck‐Loomis , S. Hao , B. Z. Yeung , W. M. Mauck III , P. Smibert , R. Satija , Genome Biol. 2018, 19, 224. 30567574 10.1186/s13059-018-1603-1 PMC6300015

[42] E. P. Mimitou , A. Cheng , A. Montalbano , S. Hao , M. Stoeckius , M. Legut , T. Roush , A. Herrera , E. Papalexi , Z. Ouyang , R. Satija , N. E. Sanjana , S. B. Koralov , P. Smibert , Nat. Methods 2019, 16, 409. 31011186 10.1038/s41592-019-0392-0 PMC6557128

[43] a) T. Wang , J. J. Wei , D. M. Sabatini , E. S. Lander , Science 2014, 343, 80; 24336569 10.1126/science.1246981 PMC3972032

[44] a) D. Feldman , L. Funk , A. Le , R. J. Carlson , M. D. Leiken , F. Tsai , B. Soong , A. Singh , P. C. Blainey , Nat. Protoc. 2022, 17, 476; 35022620 10.1038/s41596-021-00653-8 PMC9654597

[45] R. de Groot , J. Luthi , H. Lindsay , R. Holtackers , L. Pelkmans , Mol. Syst. Biol. 2018, 14, e8064. 29363560 10.15252/msb.20178064 PMC5787707

[46] a) C. Larsson , I. Grundberg , O. Soderberg , M. Nilsson , Nat. Methods 2010, 7, 395; 20383134 10.1038/nmeth.1448

[47] a) M. J. Lawson , D. Camsund , J. Larsson , O. Baltekin , D. Fange , J. Elf , Mol. Syst. Biol. 2017, 13, 947; 29042431 10.15252/msb.20177951 PMC5658705

[48] E. C. Wheeler , A. Q. Vu , J. M. Einstein , M. DiSalvo , N. Ahmed , E. L. Van Nostrand , A. A. Shishkin , W. Jin , N. L. Allbritton , G. W. Yeo , Nat. Methods 2020, 17, 636. 32393832 10.1038/s41592-020-0826-8 PMC7357298

[49] A. L. Yenkin , J. C. Bramley , C. L. Kremitzki , J. E. Waligorski , M. J. Liebeskind , X. E. Xu , V. D. Chandrasekaran , M. A. Vakaki , G. W. Bachman , R. D. Mitra , J. D. Milbrandt , W. J. Buchser , Commun. Biol. 2022, 5, 1128. 36284160 10.1038/s42003-022-04089-y PMC9596453

[50] S. Parvez , C. Herdman , M. Beerens , K. Chakraborti , Z. P. Harmer , J. J. Yeh , C. A. MacRae , H. J. Yost , R. T. Peterson , Science 2021, 373, 1146. 34413171 10.1126/science.abi8870 PMC9083377

[51] B. Haley , F. Roudnicky , Cancer Cell 2020, 38, 31. 32442401 10.1016/j.ccell.2020.04.006

[52] R. A. Panganiban , H. R. Park , M. Sun , M. Shumyatcher , B. E. Himes , Q. Lu , Proc. Natl. Acad. Sci. USA 2019, 116, 13384. 31213543 10.1073/pnas.1906275116 PMC6613086

[53] a) J. A. Morris , Z. Daniloski , J. Domingo , T. Barry , M. Ziosi , D. A. Glinos , S. Hao , E. P. Mimitou , P. Smibert , K. Roeder , E. Katsevich , T. Lappalainen , N. E. Sanjana , *bioRxiv* 2021, 2021.04.07.438882;

[54] C. Alda‐Catalinas , D. Bredikhin , I. Hernando‐Herraez , F. Santos , O. Kubinyecz , M. A. Eckersley‐Maslin , O. Stegle , W. Reik , Cell Syst. 2020, 11, 25. 32634384 10.1016/j.cels.2020.06.004 PMC7383230

[55] N. Liscovitch‐Brauer , A. Montalbano , J. Deng , A. Mendez‐Mancilla , H. H. Wessels , N. G. Moss , C. Y. Kung , A. Sookdeo , X. Guo , E. Geller , S. Jaini , P. Smibert , N. E. Sanjana , Nat. Biotechnol. 2021, 39, 1270. 33927415 10.1038/s41587-021-00902-x PMC8516442

[56] R. Lopes , K. Sprouffske , C. Sheng , E. C. H. Uijttewaal , A. E. Wesdorp , J. Dahinden , S. Wengert , J. Diaz‐Miyar , U. Yildiz , M. Bleu , V. Apfel , F. Mermet‐Meillon , R. Krese , M. Eder , A. V. Olsen , P. Hoppe , J. Knehr , W. Carbone , R. Cuttat , A. Waldt , M. Altorfer , U. Naumann , J. Weischenfeldt , A. deWeck , A. Kauffmann , G. Roma , D. Schubeler , G. G. Galli , Sci. Adv. 2021, 7, eabf5733. 34215580 10.1126/sciadv.abf5733 PMC11057712

[57] T. M. Norman , M. A. Horlbeck , J. M. Replogle , A. Y. Ge , A. Xu , M. Jost , L. A. Gilbert , J. S. Weissman , Science 2019, 365, 786. 31395745 10.1126/science.aax4438 PMC6746554

[58] a) Z. Daniloski , T. X. Jordan , H. H. Wessels , D. A. Hoagland , S. Kasela , M. Legut , S. Maniatis , E. P. Mimitou , L. Lu , E. Geller , O. Danziger , B. R. Rosenberg , H. Phatnani , P. Smibert , T. Lappalainen , B. R. tenOever , N. E. Sanjana , Cell 2021, 184, 92; 33147445 10.1016/j.cell.2020.10.030 PMC7584921

[59] a) N. M. Dräger , S. M. Sattler , C. T.‐L. Huang , O. M. Teter , K. Leng , S. H. Hashemi , J. Hong , G. Aviles , C. D. Clelland , L. Zhan , J. C. Udeochu , L. Kodama , A. B. Singleton , M. A. Nalls , J. Ichida , M. E. Ward , F. Faghri , L. Gan , M. Kampmann , Nat. Neurosci. 2022, 25, 1149; 35953545 10.1038/s41593-022-01131-4 PMC9448678

[60] J. L. McFaline‐Figueroa , A. J. Hill , X. Qiu , D. Jackson , J. Shendure , C. Trapnell , Nat. Genet. 2019, 51, 1389. 31477929 10.1038/s41588-019-0489-5 PMC6756480

[61] J. A. Belk , W. Yao , N. Ly , K. A. Freitas , Y. T. Chen , Q. Shi , A. M. Valencia , E. Shifrut , N. Kale , K. E. Yost , C. V. Duffy , B. Daniel , M. A. Hwee , Z. Miao , A. Ashworth , C. L. Mackall , A. Marson , J. Carnevale , S. A. Vardhana , A. T. Satpathy , Cancer Cell 2022, 40, 768. 35750052 10.1016/j.ccell.2022.06.001 PMC9949532

[62] E. Wang , H. Zhou , B. Nadorp , G. Cayanan , X. Chen , A. H. Yeaton , S. Nomikou , M. T. Witkowski , S. Narang , A. Kloetgen , P. Thandapani , N. Ravn‐Boess , A. Tsirigos , I. Aifantis , Cell Stem Cell 2021, 28, 718. 33450187 10.1016/j.stem.2020.12.005 PMC8145876

[63] E. Papalexi , E. P. Mimitou , A. W. Butler , S. Foster , B. Bracken , W. M. Mauck III , H. H. Wessels , Y. Hao , B. Z. Yeung , P. Smibert , R. Satija , Nat. Genet. 2021, 53, 322. 33649593 10.1038/s41588-021-00778-2 PMC8011839

[64] C. Wang , T. Lu , G. Emanuel , H. P. Babcock , X. Zhuang , Proc. Natl. Acad. Sci. USA 2019, 116, 10842. 31085639 10.1073/pnas.1903808116 PMC6561216

[65] M. A. Lalli , D. Avey , J. D. Dougherty , J. Milbrandt , R. D. Mitra , Genome Res. 2020, 30, 1317. 32887689 10.1101/gr.262295.120 PMC7545139

[66] F. K. Satterstrom , J. A. Kosmicki , J. Wang , M. S. Breen , S. De Rubeis , J. Y. An , M. Peng , R. Collins , J. Grove , L. Klei , C. Stevens , J. Reichert , M. S. Mulhern , M. Artomov , S. Gerges , B. Sheppard , X. Xu , A. Bhaduri , U. Norman , H. Brand , G. Schwartz , R. Nguyen , E. E. Guerrero , C. Dias , Autism Sequencing Consortium , iPSYCH‐Broad Consortium , C. Betancur , E. H. Cook , L. Gallagher , M. Gill , et al., Cell 2020, 180, 568.31981491

[67] J. D. Wardyn , A. S. Y. Chan , A. D. Jeyasekharan , Curr. Protoc. 2021, 1, e286. 34748280 10.1002/cpz1.286

[68] W. Li , H. Xu , T. Xiao , L. Cong , M. I. Love , F. Zhang , R. A. Irizarry , J. S. Liu , M. Brown , X. S. Liu , Genome Biol. 2014, 15, 554. 25476604 10.1186/s13059-014-0554-4 PMC4290824

[69] R. M. Meyers , J. G. Bryan , J. M. McFarland , B. A. Weir , A. E. Sizemore , H. Xu , N. V. Dharia , P. G. Montgomery , G. S. Cowley , S. Pantel , A. Goodale , Y. Lee , L. D. Ali , G. Jiang , R. Lubonja , W. F. Harrington , M. Strickland , T. Wu , D. C. Hawes , V. A. Zhivich , M. R. Wyatt , Z. Kalani , J. J. Chang , M. Okamoto , K. Stegmaier , T. R. Golub , J. S. Boehm , F. Vazquez , D. E. Root , W. C. Hahn , et al., Nat. Genet. 2017, 49, 1779. 29083409 10.1038/ng.3984 PMC5709193

[70] B. Langmead , S. L. Salzberg , Nat. Methods 2012, 9, 357. 22388286 10.1038/nmeth.1923 PMC3322381

[71] A. N. Schep , B. Wu , J. D. Buenrostro , W. J. Greenleaf , Nat. Methods 2017, 14, 975. 28825706 10.1038/nmeth.4401 PMC5623146

[72] T. Stuart , A. Butler , P. Hoffman , C. Hafemeister , E. Papalexi , W. M. Mauck III , Y. Hao , M. Stoeckius , P. Smibert , R. Satija , Cell 2019, 177, 1888. 31178118 10.1016/j.cell.2019.05.031 PMC6687398

[73] F. A. Wolf , P. Angerer , F. J. Theis , Genome Biol. 2018, 19, 15. 29409532 10.1186/s13059-017-1382-0 PMC5802054

[74] C. McQuin , A. Goodman , V. Chernyshev , L. Kamentsky , B. A. Cimini , K. W. Karhohs , M. Doan , L. Ding , S. M. Rafelski , D. Thirstrup , W. Wiegraebe , S. Singh , T. Becker , J. C. Caicedo , A. E. Carpenter , PLoS Biol. 2018, 16, e2005970. 29969450 10.1371/journal.pbio.2005970 PMC6029841

[75] G. X. Zheng , J. M. Terry , P. Belgrader , P. Ryvkin , Z. W. Bent , R. Wilson , S. B. Ziraldo , T. D. Wheeler , G. P. McDermott , J. Zhu , M. T. Gregory , J. Shuga , L. Montesclaros , J. G. Underwood , D. A. Masquelier , S. Y. Nishimura , M. Schnall‐Levin , P. W. Wyatt , C. M. Hindson , R. Bharadwaj , A. Wong , K. D. Ness , L. W. Beppu , H. J. Deeg , C. McFarland , K. R. Loeb , W. J. Valente , N. G. Ericson , E. A. Stevens , J. P. Radich , et al., Nat. Commun. 2017, 8, 14049. 28091601 10.1038/ncomms14049 PMC5241818

[76] J. Duan , G. Hon , Bioinformatics 2021, 37, 4266. 33999185 10.1093/bioinformatics/btab375 PMC9502162

[77] L. Wang , Nat. Commun. 2021, 12, 6395. 34737291 10.1038/s41467-021-26682-1 PMC8568964

[78] L. Yang , Y. Zhu , H. Yu , X. Cheng , S. Chen , Y. Chu , H. Huang , J. Zhang , W. Li , Genome Biol. 2020, 21, 19. 31980032 10.1186/s13059-020-1928-4 PMC6979386

[79] B. Duan , C. Zhou , C. Zhu , Y. Yu , G. Li , S. Zhang , C. Zhang , X. Ye , H. Ma , S. Qu , Z. Zhang , P. Wang , S. Sun , Q. Liu , Nat. Commun. 2019, 10, 2233. 31110232 10.1038/s41467-019-10216-x PMC6527552

[80] J. M. Replogle , R. A. Saunders , A. N. Pogson , J. A. Hussmann , A. Lenail , A. Guna , L. Mascibroda , E. J. Wagner , K. Adelman , G. Lithwick‐Yanai , N. Iremadze , F. Oberstrass , D. Lipson , J. L. Bonnar , M. Jost , T. M. Norman , J. S. Weissman , Cell 2022, 185, 2559. 35688146 10.1016/j.cell.2022.05.013 PMC9380471

[81] a) D. B. Burkhardt , J. S. Stanley III , A. Tong , A. L. Perdigoto , S. A. Gigante , K. C. Herold , G. Wolf , A. J. Giraldez , D. van Dijk , S. Krishnaswamy , Nat. Biotechnol. 2021, 39, 619; 33558698 10.1038/s41587-020-00803-5 PMC8122059

[82] M. Lotfollahi , F. A. Wolf , F. J. Theis , Nat. Methods 2019, 16, 715. 31363220 10.1038/s41592-019-0494-8

[83] M. Kampmann , Nat. Rev. Neurol. 2020, 16, 465. 32641861 10.1038/s41582-020-0373-z PMC7484261

[84] S. Aibar , C. B. Gonzalez‐Blas , T. Moerman , V. A. Huynh‐Thu , H. Imrichova , G. Hulselmans , F. Rambow , J. C. Marine , P. Geurts , J. Aerts , J. van den Oord , Z. K. Atak , J. Wouters , S. Aerts , Nat. Methods 2017, 14, 1083. 28991892 10.1038/nmeth.4463 PMC5937676

[85] A. Gupta , P. J. Harrison , H. Wieslander , N. Pielawski , K. Kartasalo , G. Partel , L. Solorzano , A. Suveer , A. H. Klemm , O. Spjuth , I. M. Sintorn , C. Wahlby , Cytometry A 2019, 95, 366. 30565841 10.1002/cyto.a.23701 PMC6590257

[86] a) S. Banskota , A. Raguram , S. Suh , S. W. Du , J. R. Davis , E. H. Choi , X. Wang , S. C. Nielsen , G. A. Newby , P. B. Randolph , M. J. Osborn , K. Musunuru , K. Palczewski , D. R. Liu , Cell 2022, 185, 250; 35021064 10.1016/j.cell.2021.12.021 PMC8809250

[87] a) S. I. Cho , S. Lee , Y. G. Mok , K. Lim , J. Lee , J. M. Lee , E. Chung , J. S. Kim , Cell 2022, 185, 1764; 35472302 10.1016/j.cell.2022.03.039

[88] L. Tian , F. Chen , E. Z. Macosko , Nat. Biotechnol. 2022. 10.1038/s41587-022-01448-2 PMC10091579 36192637

[89] W. T. Chen , A. Lu , K. Craessaerts , B. Pavie , C. Sala Frigerio , N. Corthout , X. Qian , J. Lalakova , M. Kuhnemund , I. Voytyuk , L. Wolfs , R. Mancuso , E. Salta , S. Balusu , A. Snellinx , S. Munck , A. Jurek , J. F. Navarro , T. C. Saido , I. Huitinga , J. Lundeberg , M. Fiers , B. De Strooper , Cell 2020, 182, 976. 32702314 10.1016/j.cell.2020.06.038

[90] A. Giladi , M. Cohen , C. Medaglia , Y. Baran , B. Li , M. Zada , P. Bost , R. Blecher‐Gonen , T. M. Salame , J. U. Mayer , E. David , F. Ronchese , A. Tanay , I. Amit , Nat. Biotechnol. 2020, 38, 629. 32152598 10.1038/s41587-020-0442-2

[91] Y. Tan , W. F. Flynn , S. Sivajothi , D. Luo , S. B. Bozal , M. Dave , A. A. Luciano , P. Robson , D. E. Luciano , E. T. Courtois , Nat. Cell Biol. 2022, 24, 1306. 35864314 10.1038/s41556-022-00961-5 PMC9901845

[92] L. Garcia‐Alonso , L. F. Handfield , K. Roberts , K. Nikolakopoulou , R. C. Fernando , L. Gardner , B. Woodhams , A. Arutyunyan , K. Polanski , R. Hoo , C. Sancho‐Serra , T. Li , K. Kwakwa , E. Tuck , V. Lorenzi , H. Massalha , M. Prete , V. Kleshchevnikov , A. Tarkowska , T. Porter , C. I. Mazzeo , S. van Dongen , M. Dabrowska , V. Vaskivskyi , K. T. Mahbubani , J. E. Park , M. Jimenez‐Linan , L. Campos , V. Y. Kiselev , C. Lindskog , et al., Nat. Genet. 2021, 53, 1698. 34857954 10.1038/s41588-021-00972-2 PMC8648563

[93] a) A. Chen , S. Liao , M. Cheng , K. Ma , L. Wu , Y. Lai , X. Qiu , J. Yang , J. Xu , S. Hao , X. Wang , H. Lu , X. Chen , X. Liu , X. Huang , Z. Li , Y. Hong , Y. Jiang , J. Peng , S. Liu , M. Shen , C. Liu , Q. Li , Y. Yuan , X. Wei , H. Zheng , W. Feng , Z. Wang , Y. Liu , Z. Wang , et al., Cell 2022, 185, 1777; 35512705 10.1016/j.cell.2022.04.003

[94] A. Raj , P. van den Bogaard , S. A. Rifkin , A. van Oudenaarden , S. Tyagi , Nat. Methods 2008, 5, 877. 18806792 10.1038/nmeth.1253 PMC3126653

[95] a) K. H. Chen , A. N. Boettiger , J. R. Moffitt , S. Wang , X. Zhuang , Science 2015, 348, aaa6090; 25858977 10.1126/science.aaa6090 PMC4662681

[96] E. Bielczyk‐Maczynska , D. Sharma , M. Blencowe , P. S. Gustafsson , M. J. Gloudemans , X. Yang , I. Carcamo‐Orive , M. Wabitsch , K. J. Svensson , C. Y. Park , T. Quertermous , J. W. Knowles , J. Li , *bioRxiv* 2022, 2022.06.27.497796.

[97] W. Chen , O. Guillaume‐Gentil , P. Y. Rainer , C. G. Gabelein , W. Saelens , V. Gardeux , A. Klaeger , R. Dainese , M. Zachara , T. Zambelli , J. A. Vorholt , B. Deplancke , Nature 2022, 608, 733. 35978187 10.1038/s41586-022-05046-9 PMC9402441

[98] C. Seydel , Nat. Methods 2021, 18, 1452. 34862499 10.1038/s41592-021-01333-x

[99] M. Tajik , M. Baharfar , W. A. Donald , Trends Biotechnol. 2022, 40, 1374. 35562238 10.1016/j.tibtech.2022.04.004

[100] M. Naeem , S. Majeed , M. Z. Hoque , I. Ahmad , Cells 2020, 9, 1608. 32630835 10.3390/cells9071608 PMC7407193

[101] J. G. Doench , N. Fusi , M. Sullender , M. Hegde , E. W. Vaimberg , K. F. Donovan , I. Smith , Z. Tothova , C. Wilen , R. Orchard , H. W. Virgin , J. Listgarten , D. E. Root , Nat. Biotechnol. 2016, 34, 184. 26780180 10.1038/nbt.3437 PMC4744125

[102] J. M. Replogle , J. L. Bonnar , A. N. Pogson , C. R. Liem , N. K. Maier , Y. Ding , B. J. Russell , X. Wang , K. Leng , A. Guna , T. M. Norman , R. A. Pak , D. M. Ramos , M. E. Ward , L. A. Gilbert , M. Kampmann , J. S. Weissman , M. Jost , *bioRxiv* 2022, 2022.07.13.499814.

[103] J. Tycko , M. Wainberg , G. K. Marinov , O. Ursu , G. T. Hess , B. K. Ego , Aradhana, A. L.i , A. Truong , A. E. Trevino , K. Spees , D. Yao , I. M. Kaplow , P. G. Greenside , D. W. Morgens , D. H. Phanstiel , M. P. Snyder , L. Bintu , W. J. Greenleaf , A. Kundaje , M. C. Bassik , Nat. Commun. 2019, 10, 4063. 31492858 10.1038/s41467-019-11955-7 PMC6731277

[104] K. A. Christie , J. A. Guo , R. A. Silverstein , R. M. Doll , M. Mabuchi , H. E. Stutzman , J. Lin , L. Ma , R. T. Walton , L. Pinello , G. B. Robb , B. P. Kleinstiver , Nat. Biotechnol. 2022. 10.1038/s41587-022-01492-y PMC10023266 36203014

[105] X. Han , Z. Liu , M. C. Jo , K. Zhang , Y. Li , Z. Zeng , N. Li , Y. Zu , L. Qin , Sci. Adv. 2015, 1, e1500454. 26601238 10.1126/sciadv.1500454 PMC4643799

[106] a) K. Lee , M. Conboy , H. M. Park , F. Jiang , H. J. Kim , M. A. Dewitt , V. A. Mackley , K. Chang , A. Rao , C. Skinner , T. Shobha , M. Mehdipour , H. Liu , W. C. Huang , F. Lan , N. L. Bray , S. Li , J. E. Corn , K. Kataoka , J. A. Doudna , I. Conboy , N. Murthy , Nat. Biomed. Eng. 2017, 1, 889; 29805845 10.1038/s41551-017-0137-2 PMC5968829

[107] Y. Pan , R. Tian , C. Lee , G. Bao , G. Gibson , Biol. Methods Protoc. 2020, 5, bpaa008. 32665975 10.1093/biomethods/bpaa008 PMC7334875

[108] R. Schmidt , Z. Steinhart , M. Layeghi , J. W. Freimer , R. Bueno , V. Q. Nguyen , F. Blaeschke , C. J. Ye , A. Marson , Science 2022, 375, eabj4008. 35113687 10.1126/science.abj4008 PMC9307090

